# CEBPG‐Mediated Palmitic Acid Adaptation of Cancer‐Associated Fibroblasts Drives Metastasis of Oral Squamous Cell Carcinoma

**DOI:** 10.1002/advs.75875

**Published:** 2026-06-02

**Authors:** Yiling Duan, Yitong Li, Yufei Wu, Xiao Yang, Rui Li, Hui Zhao, Zhengjun Shang

**Affiliations:** ^1^ State Key Laboratory of Oral & Maxillofacial Reconstruction and Regeneration Hubei Key Laboratory of Stomatology Key Laboratory of Oral Biomedicine Ministry of Education School & Hospital of Stomatology Wuhan University Wuhan China; ^2^ Department of Oral and Maxillofacial‐Head and Neck Oncology School & Hospital of Stomatology Wuhan University Wuhan China; ^3^ Taikang Center for Life and Medical Sciences Wuhan University Wuhan China

**Keywords:** cancer‐associated fibroblasts, CD36, epigenetic reprogramming, organoids, palmitic acid, tumor microenvironment

## Abstract

Fatty acids (FAs) create a pro‐metastatic niche in multiple cancers, but how the tumor microenvironment (TME) counteracts FA stress remains unclear. Here, we found that the FA transporter CD36 was upregulated in myofibroblastic cancer‐associated fibroblasts (myoCAFs), where it correlated with the metastasis of oral squamous cell carcinoma (OSCC). Among five predominant FA species enriched in OSCC tissues, palmitic acid (PA) potently activated myoCAF phenotypes across 2D, 3D, and organoid co‐culture models. In vivo, PA promoted lymph node metastasis in orthotopic tumors comprising OSCC cells and CAFs, an effect abolished by *CD36* knockdown in cancer‐associated fibroblasts (CAFs). Mechanistically, PA remodeled the chromatin landscape to enhance H3K27ac occupancy at multiple genes including *ERN1* and *TMBIM6*, two stress‐adaptive regulators. Transcription of *ERN1* and *TMBIM6* in CAFs was regulated by CCAAT/enhancer‐binding protein γ (CEBPG) in an enhancer‐associated manner. Disruption of the CEBPG‐IRE1α/TMBIM6 axis attenuated myoCAFs properties and abrogated PA‐driven metastasis. Our results unveil a stromal metabolic checkpoint and establish CEBPG‐mediated stress resilience as a therapeutic target to curtail metastasis.

Significance: Palmitic acid promotes metastasis via a stromal CEBPG‐IRE1α/TMBIM6 axis, uncovering a metabolic vulnerability that offers novel therapeutic targets to inhibit oral squamous cell carcinoma.

## Graphical abstract

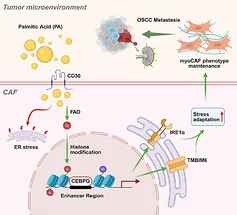

## 1 Introduction

Metabolic reprogramming is a hallmark of cancer progression [1]. To sustain rapid proliferation, cancer cells must contend with dynamic nutrient fluxes, oscillating between scarcity and overload, and develop robust mechanisms to counteract metabolic stress [2, 3]. The ability of cancer cells to adapt to these conditions is critical for their survival, growth, and metastasis. Of note, solid tumors are characterized by a complex tumor microenvironment (TME) consisting of cancer cells and stromal cells, such as cancer‐associated fibroblasts (CAFs), endothelial cells, and immune cells [4]. Although cancer cell‐intrinsic adaptive mechanisms are well‐documented, how stromal cells within the TME endure and even thrive under metabolic stresses remains poorly understood.

Fatty acids (FAs) are among the most abundant nutrients in the TME. In general, FAs interact with their transporters, such as CD36, FATPs, and ACSLs, to enter cells and serve as metabolic substrates [5, 6, 7]. Derivatives of FAs, particularly the acyl‐CoA, engage histone acylation to activate chromatin reprogramming that correlates with tumor progression [8, 9]. To date, FA uptake has been shown to drive aggressive phenotypes in lipid‐rich tissues such as breast, liver, and oral mucosa [10, 11, 12]. However, FA overload also presents a metabolic paradox: it induces endoplasmic reticulum (ER) stress and activates the unfolded protein response (UPR), while simultaneously engaging stress‐adaptive regulators in a positive feedback loop that remodels the TME [13, 14, 15, 16]. Disruption of these adaptive mechanisms may reveal targetable metabolic vulnerabilities.

Oral squamous cell carcinoma (OSCC) presents a pertinent model to investigate how FAs affect tumor development. First, a small subpopulation of CD36^+^ OSCC cells has been linked to metastasis‐initiating capacity and lipid dependency [11]. In a subsequent study comparing the oncogenic properties of common dietary fatty acids, including saturated (e.g., palmitic acid), monounsaturated (e.g., oleic acid), and polyunsaturated (e.g., linoleic acid) species, palmitic acid (PA) drastically promoted lung and lymph node metastasis in xenograft models [17]. This could be explained by the findings that PA serves as the major provider of acetyl groups for histones and thereby drives a long‐term and stable epigenetic program in cancer cells [18, 19]. Of note, the expression of CD36 in the TME was not restricted to cancer cells, as validated by scRNA‐seq analyses showing the presence of CD36^+^ stromal cells, such as CAFs and macrophages [10, 20]. However, the role of FAs in stromal cell reprogramming remains unclear. Here, we analyzed existing scRNA‐seq databases and found that the expression of CD36 correlates with the activation of myofibroblastic CAFs (myoCAFs) in multiple tumors, including head and neck cancer. Using 2D, 3D, organoid co‐culture, and orthotopic xenograft models, we dissect how FA signaling orchestrates myoCAF properties to drive OSCC progression.

## 2 Materials and Methods

### 2.1 Clinical Specimens

90 OSCC clinical specimens were obtained from surgical procedures at the Stomatological Hospital of Wuhan University. Specimens were obtained from patients with histopathologically confirmed OSCC who had not received prior radiotherapy or chemotherapy. Specimen collection and patient information (including age and TNM staging, detailed in Table S1) were approved by the Ethics Committee of the School and Hospital of Stomatology at Wuhan University (IRB‐ID: WDKQ2024A59), and written informed consent was obtained from all patients. Clinical study registration was not applicable because this was a retrospective observational study without prospective assignment of participants to interventions.

### 2.2 Cell Culture

Primary human OSCC cells and CAFs were established from tumor tissues obtained from 12 independent patients undergoing surgical resection at the School of Stomatology, Wuhan University; for each patient, one distinct cancer cell line and one CAF line were successfully derived. To minimize contamination from smooth muscle cells and pericytes, vascular‐rich regions were avoided during tumor sampling. Tissue blocks were minced and digested in tissue digestion buffer (bioGenous, K601003) at 37°C for 0.5‐2 h. The digested single cells were then cultured in T25 flasks, with CAFs adhering by the second day. After 24 h, non‐adherent cells were transferred to fresh T25 flasks pre‐coated with Matrigel (Corning, 354277) for tumor cell isolation. OSCC cells were cultured in DMEM/F12 (HyClone, SH30023.01) medium supplemented with 10% FBS (Cell‐Box, AUS‐015‐02‐S), 1× B27 (Gibco, 17504044), 1× N2 (Gibco, 17502048), 10 mm HEPES (Gibco, 15630106), and growth factors (EGF, Noggin, R‐spondin; PeproTech), while CAFs were cultured in DMEM (HyClone, SH30022.01) medium supplemented with 10% FBS. All cells were cultured at 37°C in a constant temperature environment with 5% CO_2_. Primary cell identities were confirmed by reciprocal marker characterization. OSCC cells were positive for the epithelial markers pan‐cytokeratin and E‐cadherin but negative for the fibroblast marker FAP. Conversely, CAFs expressed FAP while lacking epithelial markers. The myoCAF identity was further established by the co‐expression of α‐SMA, COL1A1, and FN1 [21, 22, 23]; this classification was restricted to cells that did not exhibit the typical morphology of vascular smooth muscle, characterized by elongated cells forming aligned, bundle‐like structures. Primary CAFs were used at passages 2–8, and primary tumor cells were used at passages 2–15 for all functional assays to minimize phenotypic drift and fibroblast senescence during in vitro culture.

### 2.3 Cultivation of Colonies and Organoids

The culture methods for fibroblast colonies, tumor organoids, and fibroblast‐attached organoids (FAOs) were described in previous literature [24, 25]. FAOs were generated by mixing CAFs and OSCC cells at a 3:1 cell ratio, followed by seeding approximately 10^6^ cells into low‐adhesion six‐well plates. Cells were cultured in suspension for 1–2 days. Subsequently, uniformly sized cell clusters were selected, embedded in Cultrex Basement Membrane Extract (R&D Systems, 3432‐005‐01), and solidified at 37°C. DMEM/F12 medium supplemented with 10% FBS, 1× N2, 1× B27, 50 ng/ml EGF was added. Growth of the FAOs was recorded using a phase‐contrast microscope (Leica DMi8) and high‐content imaging (Operetta CLS, PerkinElmer). Protrusions longer than 100 µm were defined as invasive front structures. The relative area of tumor cell invasion (ratio of day 3 to day 1) in the matrix gel and the number of invasive fronts per organoid were statistically quantified. All cells were cultured at 37°C in a constant temperature environment with 5% CO_2_.

### 2.4 Animal Models

Five‐week‐old female BALB/c‐nude mice were purchased from Shulaibao Biotechnology (Wuhan, China). Animals were fed and experiments conducted according to protocols approved by the Ethics Committee of the Stomatological Hospital, Wuhan University (S07925030D). Similar to in vitro experiments, organoids were formed by mixing CAFs (control group, CD36 knockdown group, CEBPG knockdown group, TMBIM6 knockdown group, ERN1 knockdown group, ERN1 and TMBIM6 double‐knockdown group) with OSCC cells at a 3:1 ratio. These organoids were then suspended in a 1:1 mixture of PBS and Matrigel (with or without 200 µM PA), where PA supplementation was used to model a locally lipid‐rich tumor microenvironment. Each mouse received a 50 µl injection of organoid suspension along the lingual margin, containing approximately 1.5 × 10^6^ CAFs and 5 × 10^5^ OSCC cells. Mouse body weight was recorded periodically. Tumor status was assessed via In vivo optoacoustic imaging on the sixth and tenth days. Optoacoustic imaging was performed using the In vivo High‐Speed Optoacoustic Microscopy System (TomoWave, China). At two weeks post‐injection, mice were euthanized, and tongues and cervical lymph nodes were harvested. Tumor and cervical lymph node volumes were calculated as (length × width [2])/2.

### 2.5 Transfections and Chemical Treatment

Lentiviral vectors expressing shCD36, and oeTMBIM6 were purchased from Genechem (Shanghai, China). Additionally, lentiviruses expressing the fluorescent proteins GFP and mCherry were used to label CAFs and OSCC cells with fluorescence, facilitating their visualization during growth in the matrix gel. The plasmid encoding *CEBPG* (pcDNA3.1‐CEBPG), siRNAs (in vitro and In vivo) targeting *CEBPG*, *ERN1*, and *TMBIM6* were purchased from GenePharma (Suzhou, China). Transfection of 293T cells with plasmids was performed using lipo8000 (Beyotime, C0533). siRNA transfection in CAFs was conducted using siRNA‐mate plus (GenePharma, G04026) or invivo‐siRNA mate (GenePharma, G04029). Lentiviral transfection in CAFs utilized HitransG P (Genechem, REVG005). All procedures followed the manufacturers' protocols. Palmitic acid (PA, Kunchuang Biotechnology, KC001), stearic acid (SA, Kunchuang Biotechnology, KC007), oleic acid (OA, Kunchuang Biotechnology, KC005), linoleic acid (LA, Kunchuang Biotechnology, KC009), and arachidonic acid (AA, Kunchuang Biotechnology, KC014) were used for cell treatment. PA was used at a final concentration of 50 µM for all 2D in vitro assays. This concentration was selected to approximate the lower physiological threshold of PA in human plasma (∼75‐180 µM, derived from 300–600 µM total free FAs) [26, 27, 28]. This dosage is consistent with previously reported experimental concentrations (50‐300 µM) [17] and was validated by our cytotoxicity assays. Samples were collected and analyzed after 24–48 h. When culturing organoids in matrix gel, the concentration was adjusted to 200 µM. The localized PA‐enriched state resulting from this dosage compensates for the diffusion gradients and matrix sequestration characteristic of 3D models, aligning the effective cellular exposure with the 50–100 µM baseline established for 2D assays [29, 30, 31, 32]. Treatment of CAFs with 50 µM sulfosuccinimidyl oleate sodium (SSO, MedchemExpress, HY‐112847A) or 0.05 µg/ml anti‐CD36 antibody FA6‐152 (Abcam, ab17044) for 24 h enables binding to CD36 and inhibits its transport of fatty acids into cells. Treatment of CAFs with 1 µM KIRA6 (Aladdin, K414099) for 24 h inhibits IRE1α phosphorylation. Treatment with 10 µM IXA4 (MedChemExpress, HY‐139214) for 24 h promotes IRE1α phosphorylation. Sequences of the siRNA are provided in Table S2.

### 2.6 Reverse Transcription PCR and Quantitative PCR

RNA was extracted using the conventional Trizol method. RNA was reverse transcribed into cDNA using the HiScript III RT SuperMix for qPCR kit (Vazyme, R323‐01). qPCR experiments were performed using the ChamQ SYBR qPCR Master Mix (Vazyme, Q311‐02) on a LightCycler 480 Instrument II (Roche, CH). Primer sequences are listed in Table S3. Relative expression levels were calculated using the 2^−ΔΔCt^ method, normalized to *GAPDH* as an internal control, to detect changes in gene expression.

### 2.7 Western Blot

Proteins were extracted using RIPA Lysis Buffer (Beyotime, P0013B), separated by SDS‐PAGE, transferred to PVDF membranes (Millipore, ISEQ00010), and incubated with antibodies. Primary antibody information is listed in Table S4. Secondary antibodies (PMK‐014‐090 M, PMK‐014‐091 M, PMK053M) were purchased from BIOPRIMACY. Blots were treated with ultra‐sensitive ECL chemiluminescent substrate (Biosharp, BL520B) and developed using the Odyssey FC Dual Mode Imaging System (LI‐COR Biosciences, USA).

### 2.8 Immunocytochemistry and Immunofluorescence

CAFs were washed with PBS, fixed with 4% paraformaldehyde, permeabilized with 0.2% Triton X‐100, blocked with 5% BSA, and incubated with primary antibody overnight. Fluorescent secondary antibody was incubated at room temperature for 1 h. The antibodies used are listed in Table S4. Slides were mounted with antifade mounting medium with DAPI (Beyotime, P0131). For tissue immunofluorescence (IF) or immunohistochemistry (IHC), tissue sections require overnight incubation with primary antibody, followed by incubation with fluorescent secondary antibody or HRP‐conjugated secondary antibody at room temperature for 1 h. Additionally, IHC requires DAB staining, hematoxylin counterstaining, and neutral resin mounting. Fluorescence imaging was performed using an Olympus BX53 microscope (Olympus, JP) or PANNORAMIC MIDI II (3DHISTECH, HU).

### 2.9 Flow Cytometry

CAFs were treated with vehicle, siTMBIM6, KIRA6, or siTMBIM6+KIRA6, and then stimulated with 50 µM PA for 1 or 4 days. Cells were stained using the Annexin V‐FITC/PI Apoptosis Detection Kit (Yeasen, 40302ES50) according to the manufacturer's protocol. Stained cells were analyzed within one hour using a CytoFLEX LX Flow Cytometer (Beckman, USA), and results were analyzed with FlowJo software (V10.8.1).

### 2.10 RNA‐SEQ and CUT&Tag

Correlation analysis of gene expression in tumor tissues was derived from RNA sequencing (RNA‐seq) and single‐cell RNA sequencing (scRNA‐seq) analysis of head and neck squamous cell carcinoma (HNSC) in the GEPIA3 [33] (https://gepia3.bioinfoliu.com/) and TIMER2 [34] (https://compbio.cn/timer2/) databases. Expression information for long‐chain fatty acid uptake‐related genes such as *CD36* across cell populations was sourced from the TISCH2 [35] (http://tisch.compbio.cn/) database. Single‐cell transcriptomic data of the OSCC fibroblast population were retrieved from the Gene Expression Omnibus (GEO) database (GSE215403) [36] and analyzed via the CZ CELLxGENE Discover platform [32]. Following standard quality control, normalization, and dimensionality reduction, clustering was visualized using Uniform Manifold Approximation and Projection (UMAP). Fibroblast clusters were identified using Seurat's FindAllMarkers function, based on both the top 10 differentially expressed genes per cluster and established canonical markers. Finally, CAF subtype‐associated markers were mapped onto the fibroblast UMAP to evaluate their spatial distribution and co‐expression with *CD36*. RNA from CAFs treated with or without PA for 24 h was collected for RNA‐seq. Library preparation was performed using the Optimal Dual‐mode mRNA Library Prep Kit (BGI‐Shenzhen, China), followed by PE150 sequencing on the T7 platform (BGI‐Shenzhen, China). Sequencing data were mapped to the human reference genome (GRCh38.p14). Differential gene analysis was performed using the DESeq2 package (V1.34.0) with adjusted *p* value < 0.05. Gene ontology (GO) enrichment analysis and gene set enrichment analysis (GSEA) of differentially expressed genes were conducted using the clusterProfiler package (*p* value < 0.05). CUT&Tag experiments were performed using the CUT&Tag Assay Kit (CST, 77552). Live CAFs treated with or without 50 µM PA for 1 day were harvested, and DNA was extracted according to the manufacturer's protocol. The primary antibodies used were H3K4me1 (CST, 5326), H3K27ac (CST, 8173), or IgG (CST, 2729). Secondary antibody used was Goat Anti‐Rabbit IgG (H+L) antibody (CST, 35401). Following DNA extraction, libraries were constructed using the NEBNext Ultra II DNA Library Prep Kit (NEB, E7645L) and sequenced on the Illumina Novaseq platform with PE150 setting. Sequencing data were aligned against the human reference genome (GRCh38.p14). H3K4me1 and H3K27ac peaks were detected using MACS2 (V2.1.1). Differentially accessible regions were identified with the DESeq2 package (V1.34.0). Motif enrichment analysis was performed using HOMER, and peaks were visualized with IGV (2.19.1).

### 2.11 CUT&RUN Assay

CAFs were analyzed using the CUT&RUN assay kit (CST, 86652), with the procedure strictly adhering to the manufacturer's instructions. During the experiment, Spike‐in yeast DNA was added to each sample in proportion to the cell count for normalization. Antibodies used included H3K4me1, H3K27ac, CEBPG, and IgG. Antibody information is listed in Table S4. Purified DNA served as qPCR templates to detect the presence of the *ERN1* enhancer (chr17:64,082,903‐64,081,889) and *TMBIM6* enhancer (chr12:49,750,298‐49,750,744). Relative expression levels were calculated using the 2^−ΔΔCt^ method and normalized against S. cerevisiae *ACT1* as an internal control. Primer sequences for detecting DNA fragments are listed in Table S5.

### 2.12 Enhancer Reporter Construct and Dual‐luciferase Reporter Assays

Using the JASPAR [37] database (https://jaspar.elixir.no/), we predicted binding sites for the transcription factor CEBPG on the *ERN1* enhancer fragment (chr17:64,082,903‐64,081,889) and the *TMBIM6* enhancer fragment (chr12:49,750,298‐49,750,744). The three highest‐scoring sites were selected to design mutants. The *ERN1* enhancer, *TMBIM6* enhancer, and their respective three mutants were cloned into the luciferase reporter vector pGL4.23 via standard PCR‐cloning. 293T cells were co‐transfected with the luciferase construct and pRL‐TK (Promega, E2241) under conditions with or without transfection of pcDNA3.1‐CEBPG. Detection was performed using the Dual Luciferase Reporter Assay System (Promega, E1910).

### 2.13 Gas Chromatography‐Mass Spectrometry for Fatty Acid Detection

Total free FA content in seven paired tumor and adjacent mucosa samples was quantified using the Amplex Red Free Fatty Acid Assay Kit (Beyotime, S0215S). Subsequently, three paired samples exhibiting distinct FA variations were selected for targeted quantification of 51 medium‐ and long‐chain FAs via gas chromatography‐mass spectrometry (GC‐MS). Fatty acids were extracted from 50 mg tissue with chloroform‐methanol (2:1, v/v). Lipids were derivatized to fatty acid methyl esters using 1% H_2_SO_4_ in methanol at 80°C for 30 min. After hexane extraction and washing, samples were analyzed on a Thermo Trace 1300 (Thermo Fisher Scientific, US) GC‐MS system equipped with a TG‐FAME column. Methyl salicylate was used as an internal standard for the absolute quantification of 51 medium‐ and long‐chain fatty acids. All FA concentrations were normalized by tissue weight.

### 2.14 Statistical Analysis

Data analysis was performed using GraphPad Prism (v10.4.1). Results are expressed as mean ± standard deviation (SD). Each experiment was repeated three times as independent biological replicates, and for each biological replicate, multiple technical replicates (≥3) were measured. The correlation between gene expression or protein expression in tumor tissues was analyzed using Spearman's correlation coefficient, and for analyses involving multiple correlations, the Benjamini‐Hochberg false discovery rate (FDR) procedure was applied for multiple‐testing correction. Differences in T and N staging between patient groups with high or low expression of a specific protein (e.g., CD36) in the tumor stroma were assessed using the Cochran‐Armitage trend test or Chi‐square (χ [2]) test. Other statistical comparisons were performed using Student's *t*‐test or one‐way ANOVA followed by Tukey's post hoc test. *P* < 0.05 was considered statistically significant. Asterisks denote statistical significance (*p < 0.05, **p < 0.01, *** *p* < 0.001, **** *p* < 0.0001).

### 2.15 Data Availability 

Gene expression data from HNSC and OSCC tumor tissues were obtained from the GEPIA3 (https://gepia3.bioinfoliu.com/), TIMER2 (https://compbio.cn/timer2/), TISCH2 (http://tisch.compbio.cn/), and GEO (https://www.ncbi.nlm.nih.gov/geo/) database. RNA sequencing data (GSE314457) and CUT&Tag (GSE312946) sequencing data have been submitted to the GEO database. The data generated in this study are available upon request from the corresponding author.

## 3 Results

### 3.1 Expression of CD36 in CAFs is Correlated With OSCC Metastasis

**FIGURE 1:**
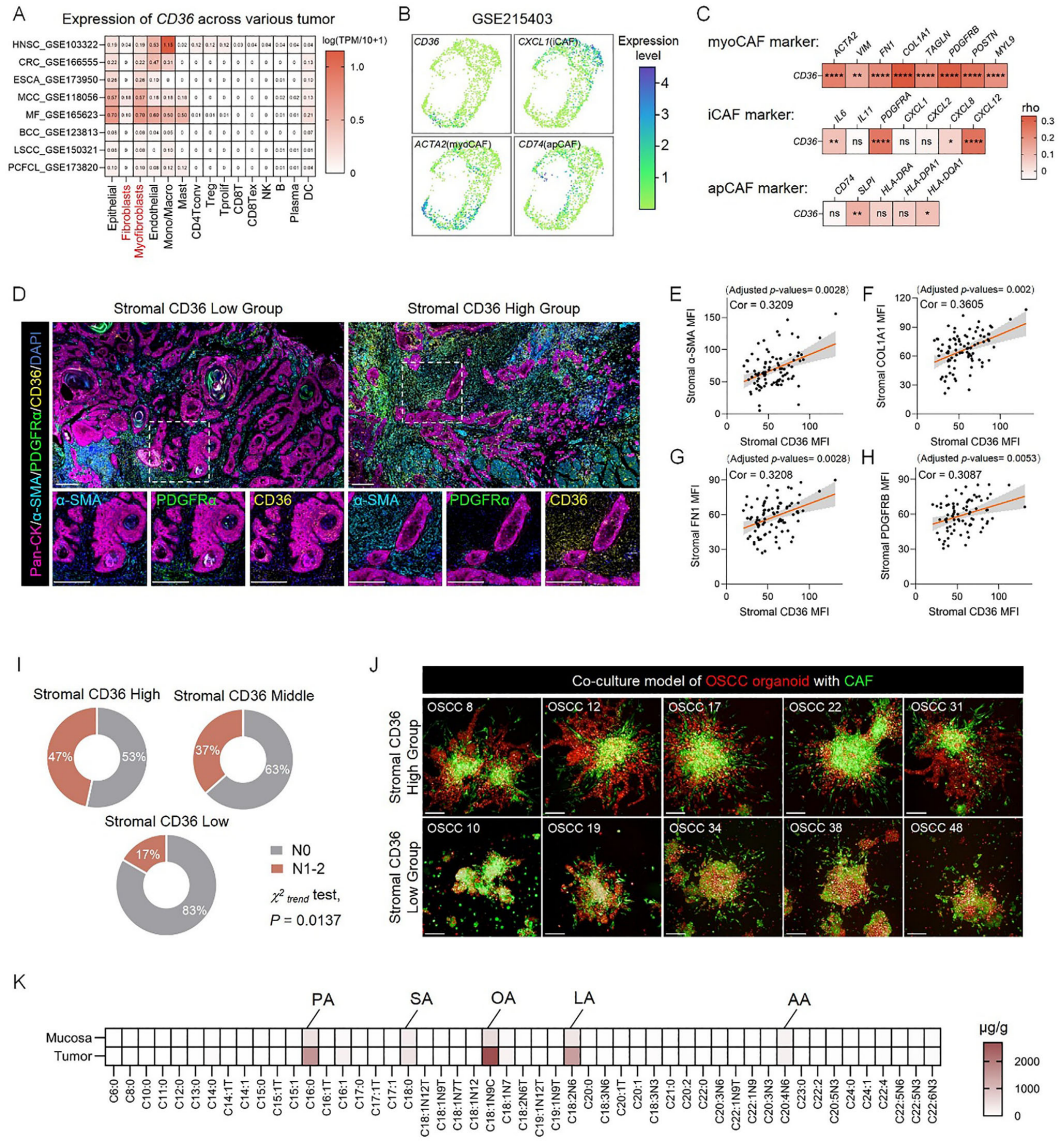
Expression of CD36 in CAFs is correlated with OSCC metastasis. (A) The expression of the *CD36* in scRNA‐seq data from the TISCH2 database for head and neck squamous cell carcinoma (HNSC), colorectal cancer (CRC), esophageal cancer (ESCA), Merkel cell carcinoma (MCC), mycosis fungoides (MF), basal cell carcinoma (BCC), lung squamous cell carcinoma (LSCC), and primary cutaneous follicular center lymphoma (PCFCL) across various cell populations. (B) UMAP plots of the fibroblast cluster from scRNA‐seq data of OSCC (GSE215403) expressing *CD36*, *ACTA2*, *CXCL1*, and *CD74*. (C) Correlation between *CD36* expression and myoCAF marker genes, iCAF marker genes, and apCAF marker genes in the GEPIA3 database. (D) Multiplex immunofluorescence staining of Pan‐CK, α‐SMA, PDGFRA, CD36, and DAPI in OSCC tissue sections from patients. (E‐H) Correlation analysis of stromal CD36 expression with stromal α‐SMA, FN1, COL1A1, and PDGFRB expression in tissue microarrays of clinical OSCC patients (n = 90), mean fluorescence intensity (MFI). (I) Comparison of N staging among stromal CD36 expression groups (high, middle and low; n = 30 per group). (J) Fluorescence images of FAOs formed by combining primary tumor cells (stained with DiI) and primary CAFs (stained with DiO) extracted from tissues with high and low stromal CD36 expression, respectively, at day 5. (K) Heatmap of 51 medium‐ and long‐chain fatty acids in tumor and mucosal tissues, detected by GC‐MS (n = 3). Statistical analysis was performed using Spearman's rank correlation for (E‐H) and the Cochran‐Armitage trend test for (I). Scale bar, 200 µm.

To investigate the role of FA metabolism in the TME, scRNA‐seq datasets from the TISCH2 database were utilized to analyze the expression of FA transporter *CD36*, *FATPs*, and *ACSLs* in multiple cancer types, including head and neck cancer, colorectal carcinomas, and so on. While cancer cells robustly expressed FA transporter *CD36*, we observed that stromal cells such as fibroblasts, endothelial cells, and monocytes also expressed abundant *CD36* (Figure 1A). Of note, upregulation of *CD36*, rather than other FA transporters (e.g., *FATPs*, *ACSLs*), correlated with both CAF infiltration and myofibroblast activation in multiple cancers including HNSC (Figure S1A–C). The UMAP plot of scRNA‐seq data (GSE215403) for the fibroblast population showed that expression of *CD36* partly overlapped with myoCAF marker *ACTA2*, but showed no correlation with markers for other CAF subtypes, such as *CXCL1* for iCAFs and *CD74* for apCAFs (Figure 1B, Figure S1D). A correlation analysis by GEPIA3 database indicated that *CD36* expression tightly correlated with all detected myoCAF marker genes, such as *FN1*, *COL1A1*, and *MYL9*, but only showed sporadic correlation with markers for iCAF and apCAF [21, 22, 23, 38] (Figure 1C). These results indicated a connection of FA metabolism with myoCAF activation. To validate this assumption, a panel of OSCC specimens (n = 90) was collected for multiplex immunofluorescence (mIF) and immunofluorescence (IF) staining. As indicated by mIF results, CD36 was expressed by both cancer and stromal cells (Figure 1D). Of note, expression of CD36 in stromal cells largely colocalized with α‐SMA (Figure 1D), a typical myoCAF marker [39]. Across all tumor specimens, protein expression of CD36 in stromal cells, rather than tumor cells, was correlated with several myoCAF markers including FN1, COL1A1, and PDGFRB (Figure 1E–H, Figure S1E–I). To evaluate the clinical relevance of stromal CD36, we quantified its mean fluorescence intensity (MFI) in all OSCC specimens. Patients were categorized into low, middle, and high groups based on stromal CD36 expression tertiles. We observed a progressive increase in the incidence of lymph node metastasis across these groups, with the highest frequency occurring in the stromal CD36‐high cohort (Figure 1I). Notably, this correlation was independent of T stage and exclusive to the stromal rather than the tumoral compartment (Figure S2A,B). To mechanistically investigate this association, primary CAFs and cancer cells were isolated from the stromal CD36‐low and CD36‐high cohorts to construct a fibroblast‐attached organoid (FAO) co‐culture model [24] (see Methods for details). Consequently, FAOs derived from the stromal CD36‐high group exhibited higher expression of CD36 in CAFs (Figure S2C) and a more aggressive morphology than those in the stromal CD36‐low group (Figure 1J, Figure S2D). In contrast, cancer cell organoids alone showed limited invasion and no appreciable differences between groups (Figure S2E,F). These results underscore the role of CD36 in myoCAF activation. In line with this, GC‐MS‐based fatty acid profiling analysis identified five predominant long‐chain FAs in OSCC tissue compared to adjacent mucosa, namely, palmitic acid (PA), stearic acid (SA), oleic acid (OA), linoleic acid (LA), and arachidonic acid (AA) (Figure 1K, Figure S3A,B), validating that the OSCC TME harbors abundant FAs. Together, these results indicate that FA metabolism is intrinsically linked to myoCAF activation in the TME.

### 3.2 CD36 in CAFs Contributes to PA‐Induced Metastasis in an Orthotopic Xenograft Model

**FIGURE 2:**
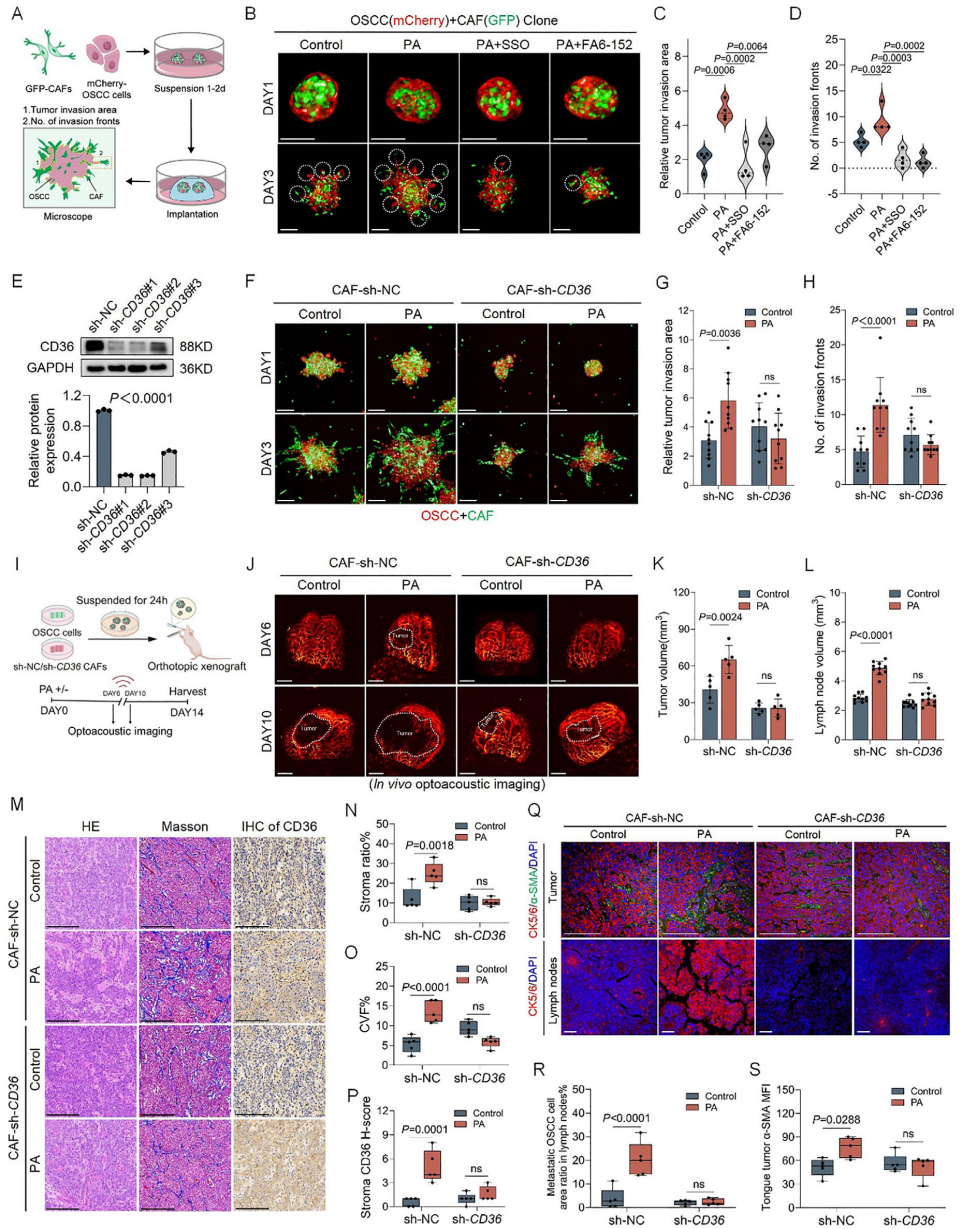
CD36 in CAFs contributes to PA‐induced metastasis in an orthotopic xenograft model. (A) Flowchart for culturing FAOs. (B) Representative fluorescence images of FAOs formed by combining mCherry‐labeled tumor cells and GFP‐labeled CAFs on days 1 and 3. (C‐D) Statistical analysis of relative tumor invasion areas (day 3 vs. day 1) and number of invasion fronts (day 3) in FAOs (n = 4). (E) Western blot validation of *CD36* knockdown efficiency in CAFs. (F) Representative fluorescence images of FAOs composed of sh‐NC‐CAFs and sh‐*CD36*‐CAFs stained with DiO and tumor cells stained with DiI on day 1 and day 3. (G‐H) Statistical analysis of relative tumor invasion areas (day 3 vs. day 1) and number of invasion fronts (day 3) in FAOs (n = 10). (I) Flowchart for establishing orthotopic xenograft models by In vivo injection of FAOs containing sh‐NC‐CAFs and sh‐*CD36*‐CAFs with or without PA treatment to BALB/c‐nude mice. (J) Representative images of optoacoustic imaging of mouse tongue tumors on day 6 and day 10. (K‐L) Statistical analysis of tumor (n = 5) and cervical lymph node volumes in mice on day 14 (n = 10). (M‐P) Representative brightfield images of H&E staining, Masson's trichrome staining, and CD36 IHC staining in mouse tongue tumor sections, along with statistical analysis of the proportion of tumor stroma, collagen area percentage, and IHC score for stromal CD36 in mouse tongue tumors (n = 5). (Q‐S) Representative images of CK5/6 and α‐SMA IF staining in tumors and CK5/6 IF staining in cervical lymph nodes. Statistical analysis of the MFI of α‐SMA in mouse orthotopic tumors and the percentage of metastatic tumor cell area in cervical lymph nodes (n = 5). All experiments were performed with at least three independent biological replicates unless otherwise specified. Data are represented as mean ± SD. Statistical differences were determined with unpaired Student's *t*‐tests and one‐way ANOVA followed by Tukey's post hoc test. Scale bars, 200 µm (B, F, M, Q), 1 mm (J).

To clarify the role of FAs in regulating myoCAF activation, five kinds of FAs enriched in OSCC, including palmitic acid (PA), stearic acid (SA), oleic acid (OA), linoleic acid (LA), and arachidonic acid (AA) were used for analysis. At the same concentration (200 µM), PA and SA more effectively promoted the morphological activation of CAFs in a 3D culture model (Figure S3C,D). Compared to SA, PA exhibited lower cytotoxicity, effectively activating CAFs while enabling their long‐term culture (Figure S3E–G). This finding is consistent with previous findings that PA, rather than other FAs, promoted OSCC development [17]. To validate the role of CD36 in mediating PA‐induced CAF activation, a specific CD36 inhibitor SSO or the neutralizing antibody FA6‐152 was added to the culture medium. As a result, addition of SSO or FA6‐152 impaired the activation of CAFs when they were co‐cultured with tumor organoids (Figure 2A–D). Importantly, control experiments revealed that treatment with PA, SSO, or FA6‐152 had no direct effect on either the viability or the invasive capacity of cancer cells cultured alone (Figure S3H, S4A–D). Subsequently, *CD36* was genetically knocked down in CAFs (Figure 2E). In the organoid co‐culture model, knockdown of *CD36* in CAFs not only impaired the activation of CAFs, but also reduced the invasion of cancer cells (Figures 2F–H, S4E,F), indicating that CD36‐mediated PA uptake contributes to myoCAF activation and the subsequent CAF‐cancer crosstalk. To test the oncogenic role of PA and CD36 in CAFs In vivo, OSCC cells and CAFs were co‐implanted into tongues of mice to generate an orthotopic xenograft model (Figure 2I). In vivo optoacoustic imaging confirmed that, PA in matrix gel accelerated the formation of xenografts in the tongue (3/5 vs 0/5 at day 6 post implantation), while knockdown of *CD36* in CAFs abrogated this effect (Figure 2J). At day 14 post implantation, all tongue xenografts (n = 5/group) and cervical lymph nodes (n = 10/group) in mice were harvested for analysis. PA increased the tumor volume and cervical lymph node volume in mice, and knockdown of *CD36* in CAFs abolished this effect (Figures 2K,L, S5A–C). IHC analysis of tongue xenografts confirmed that knockdown of *CD36* in CAFs impaired the PA‐mediated collagen deposition in tumors (Figure 2M–P), indicating that CD36‐mediated uptake of PA in CAFs enhanced extracellular matrix remodeling, a myoCAF‐related behavior [40]. Histological analyses of tissue sections, alongside qPCR analysis of human‐specific *GAPDH*, revealed that PA significantly promoted lymph node metastasis, leading to the robust formation of tumor nests (5/5 vs 1/5) confirmed by staining for the cancer cell marker CK5/6 [41] (Figures 2Q,R, S5D–F). Importantly, triple staining for CK5/6, Ki67, and TUNEL confirmed that these nests formed viable, proliferative cohesive clusters (TUNEL‐/Ki67+), indicative of established micrometastases (Figure S5G,H). Concomitantly, this enhanced metastatic spread was associated with a markedly increased abundance of myoCAFs (α‐SMA^+^ cells) in the primary orthotopic tongue xenografts (Figure 2Q,S). In line with this, knockdown of *CD36* in CAFs attenuated the above effects (Figure 2Q–S). Overall, these data demonstrate that CD36 in CAFs is essential for PA‐mediated myoCAF activation and contributes to OSCC metastasis.

### 3.3 PA Triggers Expression of Endoplasmic Reticulum Stress Adaptation Genes in myoCAFs

**FIGURE 3:**
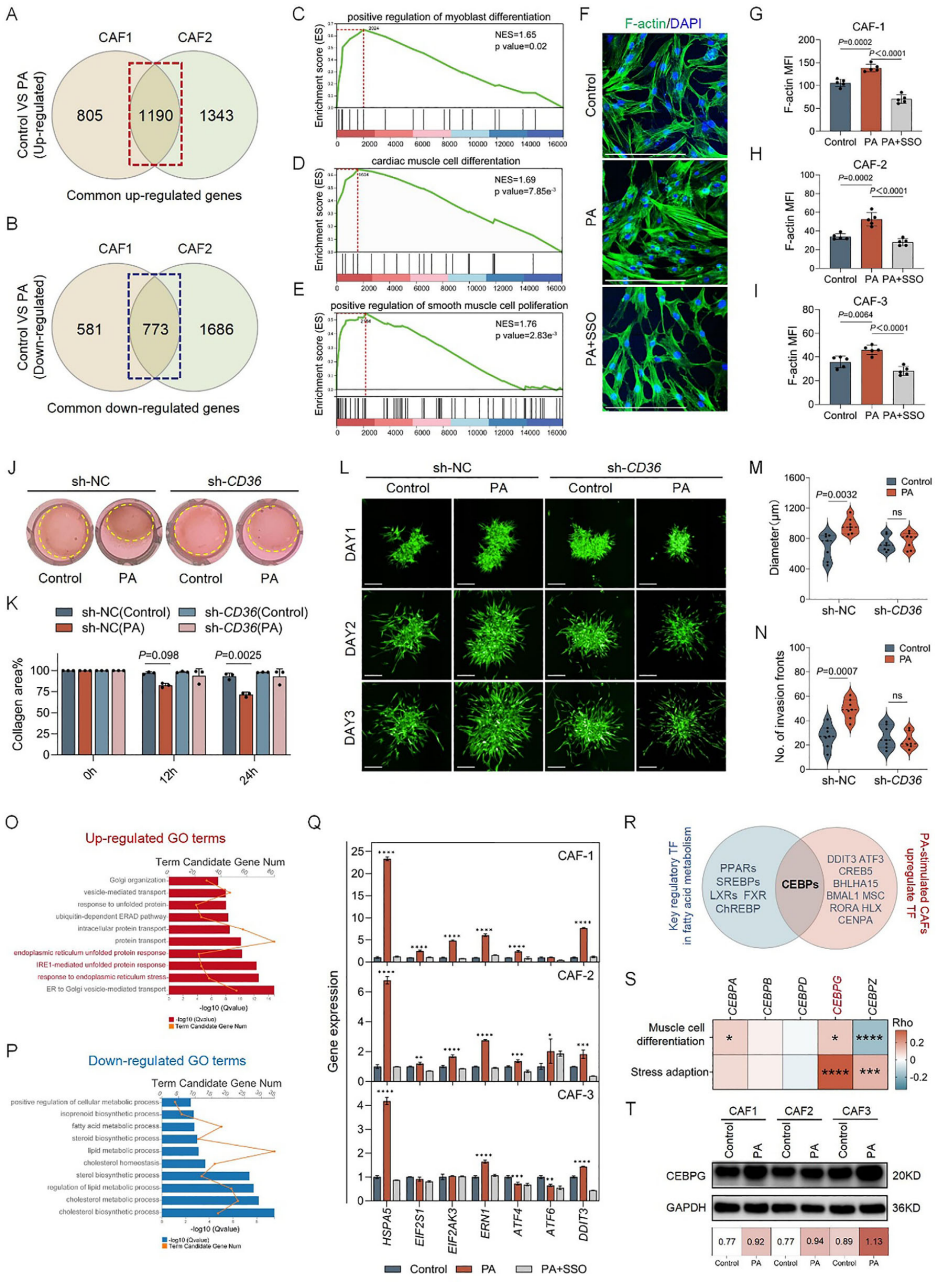
PA triggers expression of endoplasmic reticulum stress adaptation genes in myoCAFs. (A‐B) Overlap between genes upregulated or downregulated in CAF1 and CAF2 following PA treatment. (C‐E) GSEA of gene expression in CAFs treated with PA compared to control. NES, normalized enrichment score. (F‐I) F‐actin IF staining of CAFs, statistical analysis of F‐actin MFI (n = 5). (J‐K) Representative images of collagen contraction assay of CAFs at 24 h. Statistical analysis of the percentage of collagen contraction at 0, 12, and 24 h (n = 3). (L‐N) Representative fluorescence images of CAF clones growing in matrix gel from days 1 to 3. Statistical analysis of clone diameter and number of invasive fronts on day 3 (n = 7). (O‐P) GO analysis of genes co‐upregulated and co‐downregulated by CAF1 and CAF2. (Q) qPCR assay of endoplasmic reticulum stress‐related genes in CAF (n = 3–4). (R) Overlap between co‐upregulated transcription factors in CAFs following PA treatment and key transcription factors in fatty acid metabolism. (S) Correlation plots from the GEPIA3 database showing a positive correlation between *CEBPG* expression and gene sets for “Muscle cell differentiation” and “Unfolded protein response”. (T) Western blot of CEBPG expression in CAF with or without PA treatment. All experiments were performed with at least three independent biological replicates unless otherwise specified. Data are represented as mean ± SD. Statistical differences were determined with one‐way ANOVA followed by Tukey's post hoc test. Correlation analysis uses Spearman's rank correlation coefficient in (S). Asterisks denote statistical significance (*p < 0.05, **p < 0.01, ***p < 0.001, ****p < 0.0001). Scale bar, 200 µm.

To investigate how PA influences CAF phenotype, two lines of primary CAFs generated using specimens from stromal CD36‐high group were used for RNA sequencing analysis. Upon PA treatment, we identified 1,190 commonly upregulated and 773 commonly downregulated genes across both CAF lines (Figure 3A,B). To confirm the effect of PA in regulating myoCAFs properties, GSEA was further performed. The results indicated that genes related to myoblast and muscle cell differentiation were elevated (Figure 3C–E, Figure S6A). Confocal imaging analysis also validated that PA promoted the assembly of cytoskeleton (Figure 3F–I). Consistent with this, treatment with PA enhanced the myofibroblast differentiation in several experiments, including collagen contraction and 3D colony formation assays (Figure 3J–N). Importantly, the above PA‐mediated effects were rescued when CD36 in CAFs was genetically or pharmacologically inhibited (Figure 3F–N), clearly demonstrating that PA promotes myoCAFs phenotypes. GO analysis of co‐upregulated and co‐downregulated genes showed that PA promoted the expression of adaptive genes response to endoplasmic reticulum (ER) stress (Figure 3O, Figure S6A), which is in alignment with previous literature [14]. Moreover, genes related to *de novo* lipid synthesis were downregulated (Figure 3P, Figure S6B), consistent with a metabolic shift from endogenous lipid production toward reliance on exogenous PA uptake. Furthermore, qPCR analysis confirmed that PA promoted the transcription of several ER stress adaptive genes, including *HSPA5*, *DDIT3* and *ERN1* (Figure 3Q), and drug inhibition of CD36 rescued the above effects (Figure 3Q), suggesting that ER stress adaptation program was activated in CAFs upon exogenous PA treatment. To identify the key regulator of myoCAF activation, we compared the top 10 upregulated transcription factors (TFs) in our dataset with FA effector TFs that have been described in existing literature [42, 43, 44, 45, 46]. We found that CCAAT/enhancer‐binding protein (CEBP) family proteins, the TFs correlated with FA metabolism and enhancer transcription [19, 47], were broadly upregulated in our experimental model (Figure 3R). Compared with other CEBP family proteins, *CEBPG* was the only member that was upregulated in two CAF lines (Figure S6C). A correlation analysis by GEPIA3 database indicated that expression of *CEBPG* correlated with stress adaptation and myofibroblast differentiation (Figure 3S). In line with this, Western blot and qPCR analysis confirmed that PA promoted the expression of *CEBPG* across three independent CAFs (Figure 3T, Figure S6D). These results indicate that transcription of *CEBPG* is involved in PA‐induced myoCAF activation.

### 3.4 Transcription of *ERN1* and *TMBIM6* was Regulated by CEBPG via Enhancer Activation

**FIGURE 4:**
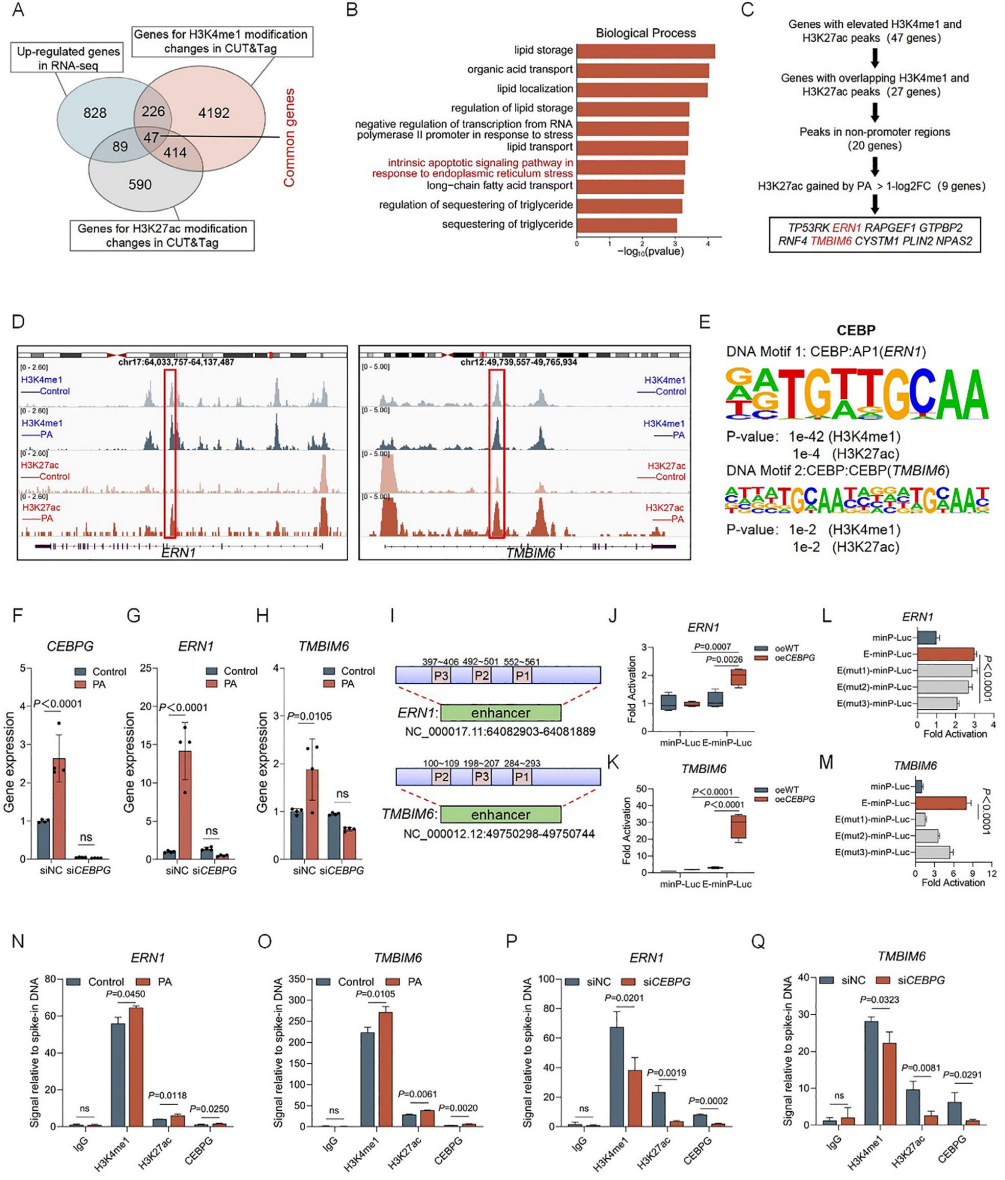
Transcription of *ERN1* and *TMBIM6* was regulated by CEBPG via enhancer activation. (A) Overlap between genes upregulated in RNA‐seq and genes showing increased H3K4me1 and H3K27ac modifications in CUT&Tag. (B) GO analysis of overlapping genes. (C) Flowchart of the strategy for identifying genes with enhancers exhibiting enhanced activity. (D) IGV tracks showing increased H3K4me1 and H3K27ac occupancy at the enhancers of *ERN1* (*p* = 0.0016; *p* < 0.0001) and *TMBIM6* (*p* < 0.0001; *p* < 0.0001) following PA treatment. (E) HOMER identification of enriched CEBP‐family binding motif from PA‐upregulated peaks, including those in *ERN1* and *TMBIM6*. (F‐H) qPCR assay of *CEBPG*, *ERN1*, and *TMBIM6* expression in CAF (n = 3–4). (I) Schematic diagram of the putative enhancer regions in the *ERN1* and *TMBIM6* genes and the predicted binding sites for the CEBPG transcription factor within these regions. (J‐M) The luciferase reporter assay for assessing the activity of *ERN1*‐E and *TMBIM6*‐E in the presence or absence of CEBPG overexpression and upon mutation of its potential CEBPG binding sites. (N‐Q) CUT&RUN‐qPCR analysis of H3K4me1, H3K27ac, and CEBPG binding at *ERN1* and *TMBIM6* enhancers upon PA treatment and following CEBPG depletion. All experiments were performed with at least three independent biological replicates unless otherwise specified. Data are represented as mean ± SD. Statistical differences were determined with unpaired Student's *t*‐tests and one‐way ANOVA followed by Tukey's post hoc test.

Given the established role of CEBPG in chromatin remodeling and enhancer activation [47, 48, 49], we sought to define its contribution to the PA‐induced activation of CAFs. Using CUT&Tag profiling of key enhancer‐associated histone marks, H3K4me1 and H3K27ac, we identified widespread chromatin alterations upon PA treatment (Figure S7A). Integration with RNA‐seq data revealed 47 genes that were coordinately upregulated at both the transcriptional and epigenetic levels (Figure 4A). Gene ontology analysis highlighted a significant enrichment for pathways involved in cellular adaptation to endoplasmic reticulum (ER) stress (Figure 4B). Among these genes, 27 exhibited co‐localized H3K4me1 and H3K27ac peaks, with 20 peaks located in non‐promoter regions. We focused on the nine genes showing the strongest PA‐induced H3K27ac enhancement, which included known ER stress regulators *ERN1* (encoding IRE1α) and *TMBIM6*, along with *TP53RK*, *RAPGEF1*, *GTPBP2*, *RNF4*, *CYSTM1*, *PLIN2*, and *NPAS2* (Figure 4C). At specific enhancer loci of *ERN1* (chr17:64,082,903‐64,081,889) and *TMBIM6* (chr12:49,750,298‐49,750,744), PA stimulation markedly increased H3K4me1 and H3K27ac occupancy (Figure 4D). Motif analysis revealed significant enrichment for CEBPG binding sites, and *CEBPG* knockdown attenuated PA‐induced upregulation of *ERN1* and *TMBIM6* at the mRNA level and reduced IRE1α and TMBIM6 protein expression (Figure S7B–D, Figure 4E–H). To determine whether CEBPG directly binds to these enhancers, we employed JASPAR‐based prediction and identified three high‐confidence CEBPG binding sites (P1‐P3) within each enhancer (Figure 4I, Figure S7E–G). Luciferase reporter assays demonstrated that enhancer fragments from *ERN1* and *TMBIM6* drove strong transcriptional activation when *CEBPG* was overexpressed (Figure 4J,K). Mutagenesis studies showed that mutation of site P3 in the *ERN1* enhancer and site P1 in the *TMBIM6* enhancer substantially abolished CEBPG‐dependent activation (Figure 4L,M), pinpointing these as functional CEBPG binding sites (*ERN1*: 397–406 bp; *TMBIM6*: 284–293 bp). Finally, CUT&RUN and qPCR assays confirmed that PA treatment enhanced the enrichment of H3K4me1, H3K27ac, and CEBPG at these enhancers, and this enhancement was abolished upon CEBPG depletion or CD36 inhibition (Figure 4N–Q, Figure S7H,I). Together, these findings establish a mechanism whereby PA drives histone modification, induces recruitment of CEBPG to specific enhancer elements of *ERN1* and *TMBIM6*, and activates transcription to facilitate ER stress adaptation in CAFs.

### 3.5 Targeting CEBPG‐IRE1α/TMBIM6 Axis Inhibits myoCAF Activation and OSCC Metastasis

**FIGURE 5:**
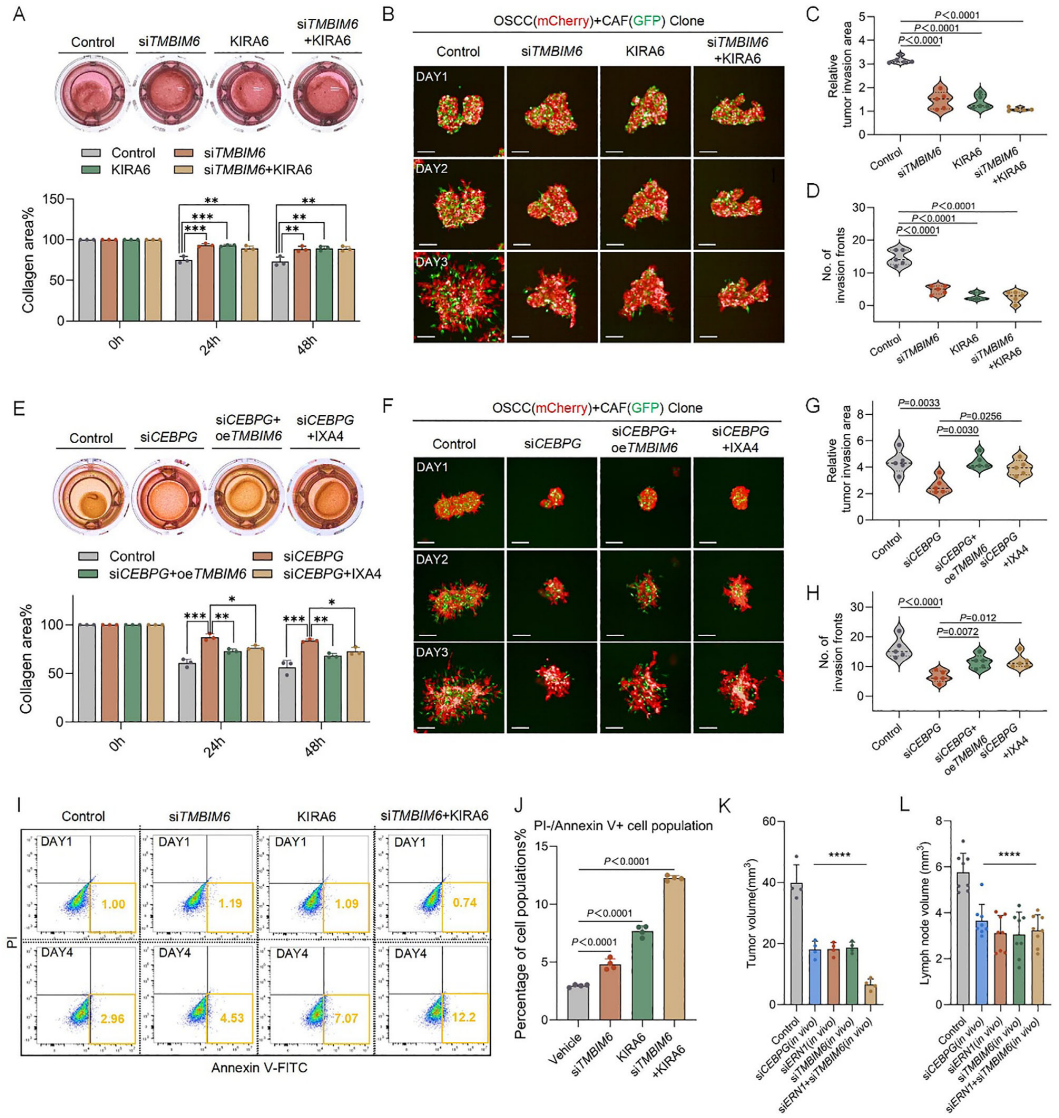
Targeting CEBPG‐IRE1α/TMBIM6 axis inhibits myoCAF activation and OSCC metastasis. (A) Representative images of collagen contraction experiments with or without KIRA6 and *TMBIM6*‐knockdown in a PA environment at 48 h. Statistical analysis of collagen contraction percentages at 0, 24, and 48 h (n = 3). (B‐D) Representative fluorescence images of FAOs composed of GFP‐CAFs with or without KIRA6 and *TMBIM6*‐knockdown treatment and mCherry‐tumor cells in a PA environment on day 1 and day 3. Statistical analysis of tumor invasion areas (day 3 vs. day 1) and number of invasion fronts (day 3) in FAOs (n = 5). (E) Representative images of collagen contraction experiments with or without *CEBPG*‐knockdown, *TMBIM6*‐overexpression and IXA4 in a PA environment at 48 h. Statistical analysis of collagen contraction percentages at 0, 24, and 48 h (n = 3). (F‐H) Representative fluorescence images of FAOs composed of GFP‐labeled CAFs with or without *CEBPG*‐knockdown, *TMBIM6*‐overexpression and IXA4 treatment and mCherry‐labeled tumor cells in a PA environment on day 1 and day 3. Statistical analysis of tumor invasion areas (day 3 vs. day 1) and number of invasion fronts (day 3) in FAOs (n = 5). (I‐J) Representative FACS plots of Annexin V staining versus PI staining in CAFs with or without KIRA6 and *TMBIM6*‐knockdown treatment after 1 day and 4 days of PA treatment. Statistical analysis of the percentage of Annexin V^+^/PI^−^ cell population (n = 4). (K‐L) Statistics on the volume of orthotopic tongue tumors and cervical lymph nodes in BALB/c‐nude mice established from organoids formed by tumor cells and PA‐treated CAFs with the indicated knockdowns (*CEBPG*, *ERN1*, *TMBIM6*, or *ERN1*/*TMBIM6* double‐knockdown). All experiments were performed with at least three independent biological replicates unless otherwise specified. Data are represented as mean ± SD. Statistical differences were determined with unpaired Student's *t*‐tests and one‐way ANOVA followed by Tukey's post hoc test. Asterisks denote statistical significance (*p < 0.05, **p < 0.01, ***p < 0.001, ****p < 0.0001). Scale bar, 200 µm.

To define the functional significance of the CEBPG‐IRE1α/TMBIM6 axis in myoCAF activation and OSCC metastasis, we established *ERN1*‐knockdown, *TMBIM6*‐knockdown, *TMBIM6*‐overexpression, and *CEBPG*‐knockdown CAF models and pharmacologically modulated IRE1α activity using KIRA6 (inhibitor) and IXA4 (activator) (Figure S8A–F). Functional assessment revealed that both *TMBIM6* knockdown and IRE1α inhibition significantly impaired CAF‐mediated collagen contraction (Figure 5A). In organoid co‐culture models, combined suppression of TMBIM6 and IRE1α markedly reduced invasive area of tumor cells and decreased the number of invasive fronts (Figure 5B–D). Consistently, knockdown of ERN1 produced inhibitory effects on collagen contraction and organoid co‐culture models comparable to those of KIRA6, further supporting the conclusion that these phenotypes are attributable to IRE1α pathway suppression rather than off‐target effects of KIRA6 (Figure S8G–K). In line with this, *CEBPG* knockdown attenuated both collagen contractility and tumor cell invasion, while *TMBIM6* overexpression or IRE1α activation with IXA4 partially restored these functional deficits (Figure 5E–H). Given that sustained ER stress signaling can trigger apoptosis, we further evaluated the protective role of this axis in CAF survival under PA challenge. Following 4 days of PA treatment, both *TMBIM6* knockdown and IRE1α inhibition increased early apoptosis (Annexin V^+^/PI^−^ cell population) in CAFs (Figure 5I,J). In vivo xenograft experiments similarly validated that inhibition of *CEBPG*, *ERN1* or *TMBIM6*, as well as the combined knockdown of *ERN1* and *TMBIM6* in CAFs all affected tumor growth (Figure 5K,L, Figure S9A–C). Collectively, these results establish that the CEBPG‐IRE1α/TMBIM6 axis safeguards CAFs against PA‐induced apoptosis and is essential for maintaining their contractile function and pro‐invasive capacity in a lipid‐rich tumor microenvironment.

### 3.6 Expression of CEBPG‐IRE1α/TMBIM6 Axis in CAFs Predicts OSCC Metastasis

**FIGURE 6:**
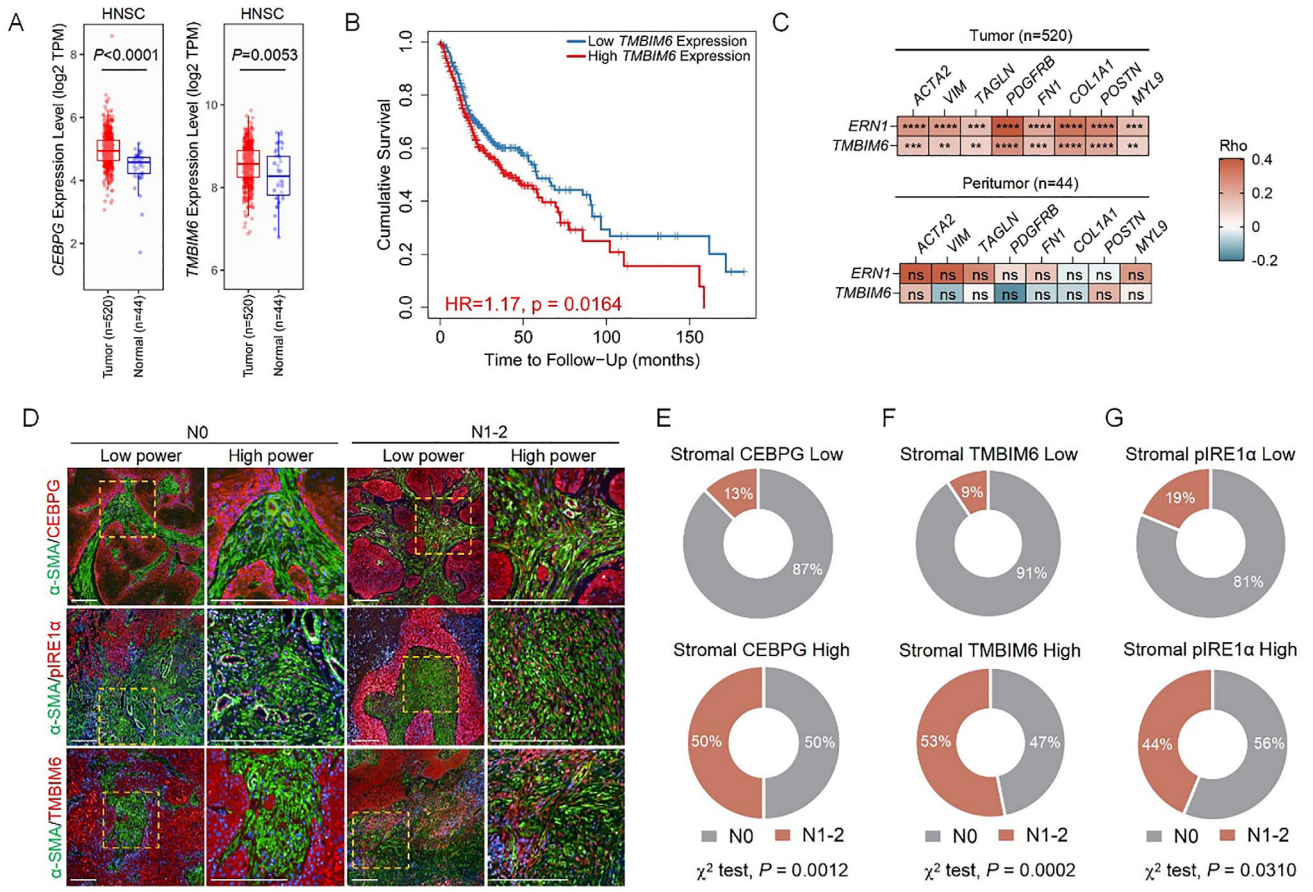
Expression of CEBPG‐IRE1α/TMBIM6 axis in CAFs predicts OSCC metastasis. (A) Gene expression of *CEBPG* and *TMBIM6* in HNSC and normal tissues within the TIMER2 database. (B) Kaplan‐Meier survival curve for *TMBIM6* gene in HNSC from the TIMER2 database. (C) Correlation between *ERN1* and *TMBIM6* gene expression and myoCAF marker genes in the GEPIA3 database. (D‐G) Representative images of IF staining for CEBPG, pIRE1α, and TMBIM6 in OSCC tumor microarrays from clinical patients. Statistical analysis and comparison of N staging between groups with high (n = 32) and low (n = 32) stromal CEBPG (E), pIRE1α (F), and TMBIM6 (G) expression. Data are represented as mean ± SD. Statistical significance was determined using Student's *t*‐test for (A), the log‐rank test for (B), Spearman's rank correlation for (C), and the Chi‐square (χ2) test for (E‐G). Asterisks denote statistical significance (*p < 0.05, **p < 0.01, ***p < 0.001, ****p < 0.0001). Scale bar, 200 µm.

To evaluate the clinical relevance of the CEBPG‐IRE1α/TMBIM6 axis, we first analyzed HNSC datasets from the GEPIA3 and TIMER2 platforms. As a result, expression of both *CEBPG* and *TMBIM6* was upregulated in tumor tissues compared to normal counterparts (Figure 6A), and the elevated *TMBIM6* expression correlated with unfavorable patient prognosis (Figure 6B). Conversely, the expression of *ERN1* mRNA showed no statistically significant difference between tumor and normal tissues, nor did it show a significant association with overall patient prognosis (Figure S9D,E). Nevertheless, correlation analysis of genes in 520 tumor and 44 normal samples demonstrated strong positive correlations of both *ERN1* and *TMBIM6* expression with established myoCAF marker genes, suggesting a potential connection between stress adaptation and myoCAF activation (Figure 6C). To validate these assumptions at the protein level, we performed IF staining to test the expression of CEBPG, IRE1α, and TMBIM6 in OSCC tissue microarrays. Clinical correlation analysis across 64 OSCC patients demonstrated that high stromal expression of CEBPG, pIRE1α, and TMBIM6 was significantly associated with lymph node metastasis (Figure 6D–G), while no such association was found for their expression in tumor cells (Figure S9F–H). Collectively, these findings establish the CEBPG‐IRE1α/TMBIM6 axis as a crucial mediator of stromal stress adaptation, myoCAF activation, and metastatic potential in patients with OSCC.

## 4 Discussion

**FIGURE 7:**
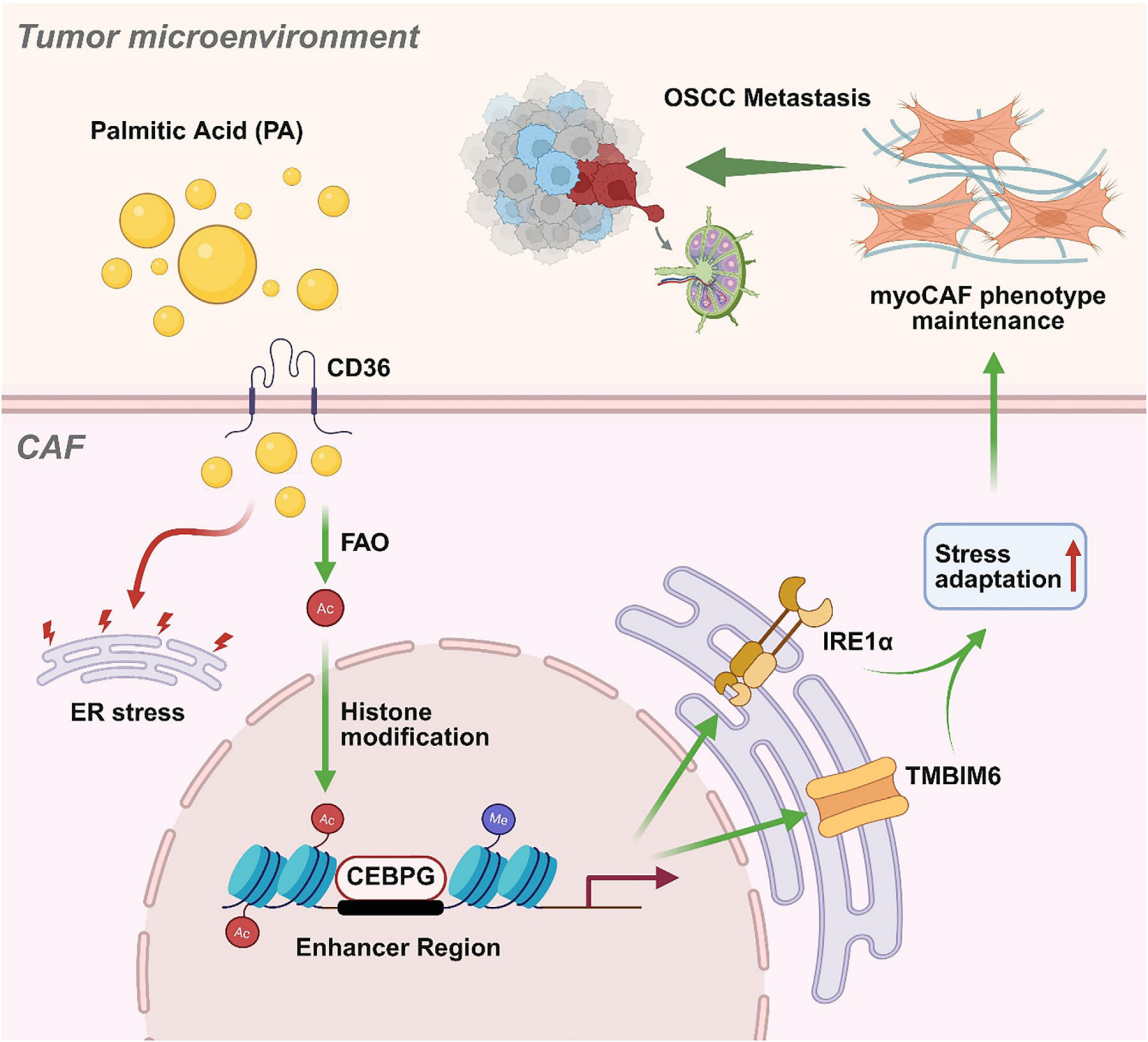
Schematic model of PA‐driven stromal reprogramming in OSCC metastasis. This study reveals a metabolic signaling axis in the OSCC microenvironment where palmitic acid (PA) drives the activation of CAFs. PA uptake triggers CEBPG‐dependent epigenetic remodeling to upregulate *ERN1* and *TMBIM6*, thereby mitigating ER stress. This adaptive program sustains CAF survival and the pro‐metastatic phenotype, establishing this pathway as a critical driver of the pro‐tumorigenic stromal niche. Image created with BioRender.com.

Our study elucidates a critical and previously unrecognized mechanism by which the lipid‐rich TME of OSCC actively promotes metastasis. We demonstrate that patient‐derived primary CAFs (Figure S10A,B) utilize the FA transporter CD36 to sense and internalize PA, triggering a CEBPG‐dependent epigenetic program that activates an IRE1α/TMBIM6‐mediated stress adaptation response. This cascade not only ensures CAF survival under conditions of lipotoxicity but also dictates their differentiation into a pro‐metastatic myoCAF phenotype, thereby establishing a direct signaling axis linking TME metabolism to stromal cell function and tumor progression (Figure 7).

A central finding of our work is the specific association of CD36 expression with the myoCAF subpopulation. While metabolic reprogramming is a recognized hallmark of cancer [1], our data transcend general observations by pinpointing CD36, out of several FA transporters, as a defining marker of activated, contractile CAFs. This specificity is crucial, as our analysis revealed that while other FA transporters like *FATP1* correlate with general CAF infiltration, only *CD36* shows unique enrichment within the pro‐metastatic myoCAF subpopulation. The strong correlation between stromal CD36 expression and myoCAF markers like ACTA2 and FN1, coupled with its association with lymph node metastasis in OSCC patients, provides compelling evidence for its functional significance. This specificity suggests that CD36 is not merely a passive conduit for fatty acid uptake but rather a key sensor that integrates metabolic cues from the TME to initiate a specific, pro‐fibrotic cellular program. Our finding that CD36‐high CAFs from patient tumors inherently exhibit a more aggressive phenotype in organoid co‐cultures further substantiates its role as a master regulator of myoCAF identity and function.

Mechanistically, we uncover that the influx of PA through CD36 initiates a profound transcriptional reprogramming in CAFs, centered around the induction of ER stress. This aligns with established knowledge that saturated fatty acids like PA can induce lipotoxicity and ER stress [14]. Notably, saturated fatty acids are uniquely potent in triggering UPR signaling and IRE1α activation due to their distinct biophysical properties (Figure S10C), whereas unsaturated lipids are preferentially esterified into neutral lipid pools to buffer cellular stress [12, 14]. This mechanistic divergence explains our observation that PA, but not oleic or linoleic acid, selectively drives the adaptive response and myoCAF differentiation independent of simple differences in cellular uptake. However, our key discovery is the identification of the transcription factor CEBPG as the pivotal mediator of the adaptive response to this stress. While the CEBP family is broadly implicated in differentiation and metabolic regulation [50], our data uniquely position CEBPG as the specific and consistently upregulated member that orchestrates the subsequent pro‐survival and pro‐fibrotic program in CAFs. The concurrent downregulation of *de novo* lipid synthesis pathways strongly supports a model where CAFs switch from endogenous production to exogenous lipid acquisition, a metabolic flexibility that likely provides a selective advantage within the nutrient‐competitive TME.

The novelty of our study is further underscored by the precise dissection of the CEBPG‐driven epigenetic mechanism. By integrating transcriptomic and CUT&Tag profiling, we demonstrate that PA enhances fatty acid oxidation (FAO) and increases intracellular acetyl‐CoA levels in CAFs (Figure S10D–G). While the exact regulatory coupling between FAO‐derived acetyl‐CoA and CEBPG‐mediated epigenetic remodeling remains to be fully elucidated, we postulate that this metabolic shift may facilitate the activation of *ERN1* and *TMBIM6* enhancers. This metabolic‐epigenetic interplay triggers a sophisticated, two‐pronged cellular defense strategy. Specifically, the activation of IRE1α, a core sensor of the unfolded protein response (UPR), enables CAFs to adapt to and resolve ER stress, while the upregulation of TMBIM6, a potent anti‐apoptotic protein resident in the ER membrane, ensures their survival [51, 52]. This CEBPG‐IRE1α/TMBIM6 axis effectively transforms a potentially lethal metabolic stressor (PA) into a differentiation signal, reprogramming CAFs into robust, long‐lived myoCAFs capable of sustained ECM remodeling and invasion support.

Our findings significantly advance the current understanding of stromal‐tumor metabolic crosstalk. Previous seminal work has highlighted the importance of CAF‐derived metabolites, often through a “reverse Warburg effect” involving lactate or amino acid exchange, in fueling cancer cell proliferation [53, 54]. Our study complements and expands this paradigm by demonstrating that CAFs are not just metabolic providers but also critical metabolic sensors. We shift the focus from glycolysis to fatty acid metabolism as a primary driver of the pro‐metastatic CAF phenotype. While the role of CD36 in promoting metastasis by enhancing fatty acid uptake in cancer cells has been elegantly shown [17], our work reveals its non‐redundant and equally critical function within the stromal compartment. We propose a model where cancer cells create a lipid‐rich niche that is then hijacked by CD36‐expressing CAFs, which in turn remodel the stroma to facilitate the invasion and dissemination of those same cancer cells, creating a vicious, self‐reinforcing metastatic cycle.

The clinical implications of our findings are substantial. The direct correlation of the CD36‐CEBPG‐IRE1α/TMBIM6 axis components with myoCAF abundance and poor prognosis in OSCC patients suggests their strong potential as prognostic biomarkers. Furthermore, while we confirmed that this stromal correlation is independent of primary tumor extent (T stage) (Figure S2A), incorporating additional variables like tumor thickness and grade in future cohorts will further refine its predictive value. Beyond its prognostic potential, this entire pathway presents a landscape of actionable targets for therapeutic intervention. Inhibiting CD36 on stromal cells, for instance, could disrupt the initial sensing of the lipid‐rich environment, effectively rendering CAFs unresponsive to the pro‐metastatic signal. Inhibition of IRE1α or targeting TMBIM6 could undermine the adaptive mechanisms of CAFs, rendering them susceptible to PA‐induced apoptosis and thereby dismantling the pro‐metastatic stromal architecture. Such a strategy, aimed at reprogramming the supportive stroma rather than directly killing tumor cells, represents a promising therapeutic avenue to overcome resistance and prevent metastatic recurrence.

While our study establishes a robust mechanistic framework for stromal metabolic regulation in OSCC, several avenues remain open for future investigation. First, our assessment of fatty acid concentrations relied on systemic plasma levels. Given the profound technical challenges inherent in isolating and analyzing tumor interstitial fluid (TIF), plasma composition serves as a reliable yet approximate proxy for the TME. Future studies incorporating direct TIF analysis or stable isotope tracing will be essential to precisely define the metabolic landscape and the ultimate sources of intratumoral PA (e.g., dietary intake, adipocyte lipolysis, or cancer cell secretion). Second, while our reliance on immunocompromised models allowed us to isolate the CAF‐tumor crosstalk, it precluded the analysis of immune cell interplay. Since *CD36* is also expressed in monocytes and endothelial cells, a compelling next step is to evaluate how this metabolic axis shapes the broader immune and vascular microenvironment in immunocompetent models. Third, while our transcriptomic and epigenetic profiling identified the core CEBPG‐driven mechanism, the field would benefit from further proteomic approaches to resolve the dynamic post‐translational activation of proteins like IRE1α. Finally, although our data suggest that PA is a potent activator, the OSCC TME contains a complex milieu of lipids; notably, we observed that PA‐driven FAO and invasion persist even in the presence of oleic acid (Figure S10H–I). Nevertheless, future research should elucidate the combinatorial effects and competitive dynamics of these diverse lipid species on CAF reprogramming. These future inquiries will undoubtedly refine our understanding of the metabolic ecosystem within the TME and facilitate the translation of these findings into clinical practice.

In conclusion, our study delineates a defined signaling pathway from a specific TME metabolite to a distinct epigenetic program that dictates stromal cell fate and promotes cancer metastasis. We establish that the PA‐CD36‐CEBPG‐IRE1α/TMBIM6 axis functions as a critical rheostat for lipo‐adaptation and myoCAF activation in OSCC. By uncovering this stromal vulnerability, our work provides a compelling rationale for targeting metabolic crosstalk within the TME as a novel therapeutic strategy to abrogate cancer progression.

## Author Contributions

**Y. Duan** contributed to data curation, software development, formal analysis, validation, investigation, visualization, methodology, and writing of the original draft. **Y. Li** contributed to data curation, formal analysis, validation, investigation, and methodology. **Y. Wu** contributed to formal analysis, validation, investigation, and methodology. **X. Yang** contributed to data curation, validation, investigation, and methodology. **R. Li** contributed to data curation, validation, investigation, and methodology. **H. Zhao** contributed to conceptualization, supervision, funding acquisition, writing of the original draft, and review and editing of the manuscript. **Z. Shang** contributed to conceptualization, resources, supervision, funding acquisition, project administration, and review and editing of the manuscript.

## Conflicts of Interest

The authors declare no conflicts of interest.

## Supporting information




**Supporting File**: advs75875‐sup‐0001‐SuppMat.docx.

## Data Availability

The data that support the findings of this study are openly available in Gene Expression Omnibus at https://www.ncbi.nlm.nih.gov/geo, reference number GSE314457 and GSE312946.
