# Supplementary material for: CEBPG‐Mediated Palmitic Acid Adaptation of Cancer‐Associated Fibroblasts Drives Metastasis of Oral Squamous Cell Carcinoma
Source: Adv Sci (Weinh). 2026 Jun 2:e75875. Online ahead of print. doi: 10.1002/advs.75875 (PMC13336018; doi:10.1002/advs.75875)
Supplement: Supplementary file 1 — Supporting file: advs75875‐sup‐0001‐SuppMat.docx [file ADVS-9999-e75875-s001.docx]

**Supplementary information**

**CEBPG-mediated** **palmitic acid adaptation of cancer-associated fibroblasts drives metastasis of oral squamous cell carcinoma**

Yiling Duan^1#^, Yitong Li^1#^, Yufei Wu^1^, Xiao Yang^1^, Rui Li^1^, Hui Zhao^1,2*^, Zhengjun Shang^1,2,3*^

^1^State Key Laboratory of Oral & Maxillofacial Reconstruction and Regeneration, Key Laboratory of Oral Biomedicine Ministry of Education, Hubei Key Laboratory of Stomatology, School & Hospital of Stomatology, Wuhan University, Wuhan, China

^2^Department of Oral and Maxillofacial-Head and Neck Oncology, School & Hospital of Stomatology, Wuhan University, Wuhan, China

^3^Taikang Center for Life and Medical Sciences, Wuhan University, Wuhan, China

**Running title:** CEBPG enables stress adaptation of metastasis-related CAF

^#^**Contributed equally;**

**Corresponding Authors:**

*Correspondence 1: Zhengjun Shang, MD., Ph.D.; Department of Oral and Maxillofacial-Head and Neck Oncology, School of Stomatology-Hospital of Stomatology, Wuhan University, 237 Luoyu Rd., Hongshan District, Wuhan 430079, China. E-mail: [shangzhengjun@whu.edu.cn](mailto:shangzhengjun@whu.edu.cn)

*Correspondence 2: Hui Zhao, MD., Ph.D.; Department of Oral and Maxillofacial-Head and Neck Oncology, School of Stomatology-Hospital of Stomatology, Wuhan University, 237 Luoyu Rd., Hongshan District, Wuhan 430079, China. E-mail: zhaohui0824@whu.edu.cn

**Keywords:** Cancer-associated fibroblasts; Tumor microenvironment; Palmitic acid; Epigenetic reprogramming; CD36

**Supplementary figures and figure legends**

**
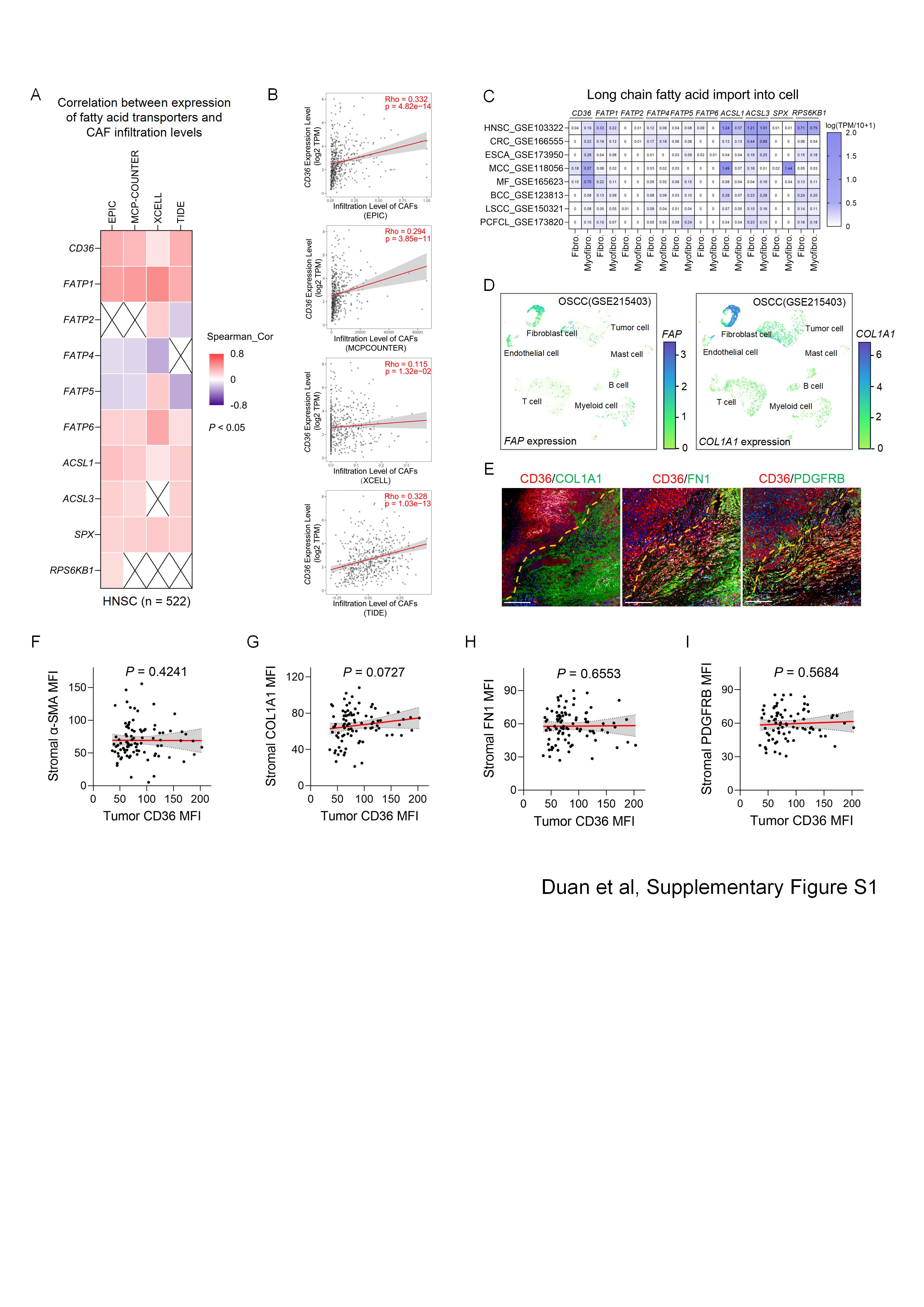
Supplementary Figure S1. Stromal CD36 expression is associated with CAF infiltration (related to Figure 1).**

(A-B) Correlation between *CD36, FATPs* and *ACSLs* expression and the infiltration level of CAFs in HNSC using the TIMER2 database. (C) The expression profiles of genes associated with long-chain fatty acid uptake in fibroblast and myofibroblast populations across scRNA-seq data from head and neck squamous cell carcinoma (HNSC), colorectal cancer (CRC), esophageal cancer (ESCA), Merkel cell carcinoma (MCC), mycosis fungoides (MF), basal cell carcinoma (BCC), lung squamous cell carcinoma (LSCC), and primary cutaneous follicular center lymphoma (PCFCL) in the TISCH2 database. (D) Feature plots showing the expression of *FAP* and *COL1A1* in the OSCC scRNA-seq dataset GSE215403. (E) Representative immunofluorescence (IF) images of CD36 co-staining with COL1A1, FN1, and PDGFRB in OSCC tissue sections. (F-I) Correlation analysis of tumor parenchymal CD36 expression with stromal α-SMA, COL1A1, FN1 and PDGFRB expression in tissue microarrays from clinical OSCC patients (n = 90), mean fluorescence intensity (MFI). Correlation analysis uses Spearman's rank correlation coefficient (A-B, F-I). Scale bar, 200 μm.

**
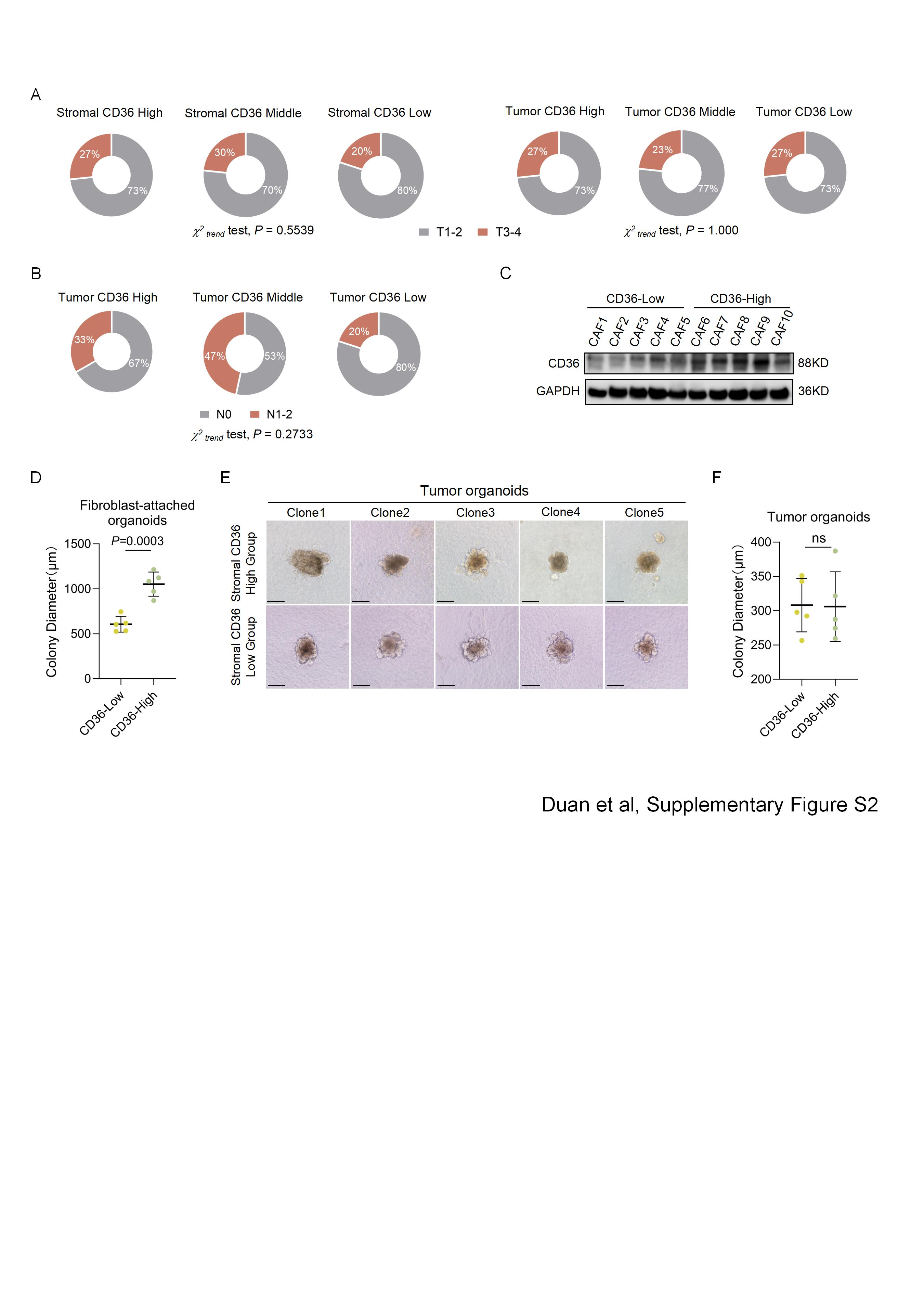
Supplementary Figure S2. Clinical relevance and functional validation of tumor and stromal CD36 in OSCC (related to Figure 1).**

(A) Comparison of T staging across high, middle and low CD36 expression groups in tumor and stromal compartments (n = 30 per group). (B) Comparison of N staging across high, middle and low CD36 expression groups in tumor compartments (n = 30 per group). (C) Western blot validation of CD36 expression in CAFs extracted from stromal CD36-high and stromal CD36-low tissues. (D) Statistical analysis of the diameter on day 5 for FAOs derived from stromal CD36-high and stromal CD36-low tissues (n = 5). (E) Representative bright-field images of tumor organoids derived from stromal CD36-high and stromal CD36-low tissues on day 5. (F) Statistical analysis of tumor organoid diameters (n = 5). All experiments were performed with at least three independent biological replicates unless otherwise specified. Data are represented as mean ± SD. Statistical differences were determined with unpaired Student's t-tests (D, F). The Cochran-Armitage test for trend was used for (A, B). Scale bar, 200 μm.

**
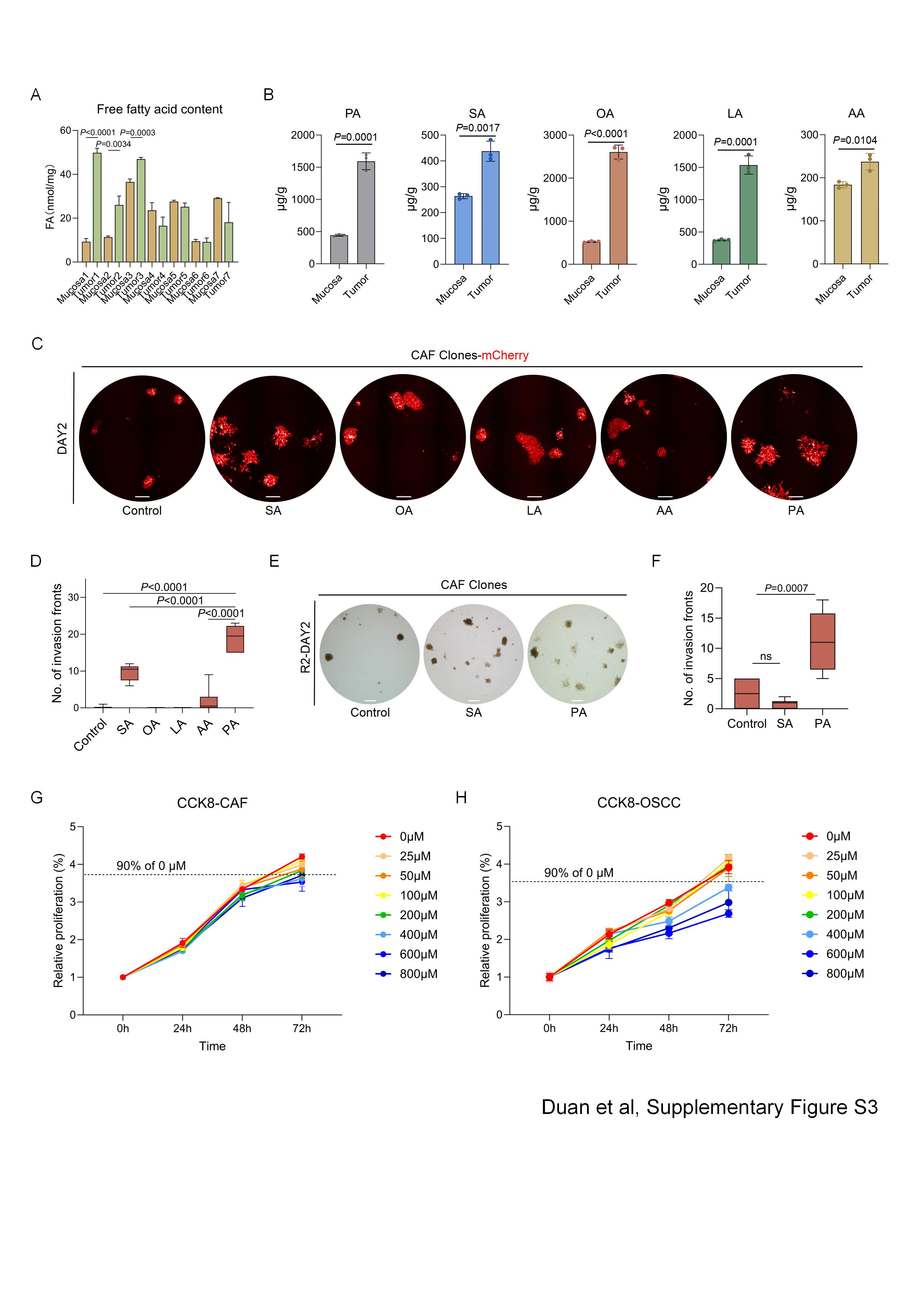
Supplementary Figure S3. PA induces a stable, pro-invasive myoCAF phenotype (related to Figure 2).**

(A) Free fatty acid concentration in tumor tissue and normal mucosa (n = 7). (B) Quantification of five predominant fatty acids (PA, SA, OA, LA, and AA) in mucosa and tumor tissues (n = 3). (C-D) Representative fluorescence images of mCherry-labeled CAF-formed colonies on day 2 after stimulation with stearic acid (SA), oleic acid (OA), linoleic acid (LA), arachidonic acid (AA), and palmitic acid (PA). Statistical analysis of invasion fronts in CAF colonies on day 2 (n = 5). (E-F) Phenotypic stability assay. To assess if the PA-induced phenotype was stable, primary colonies (from C) were dissociated and re-plated for a secondary colony formation assay without further stimulation. (E) Representative images and (F) quantification of invasion fronts show that CAFs previously treated with PA retained their invasive phenotype (n = 5). (G-H) CCK-8 assay of CAFs and OSCC cells treated with PA at concentrations ranging from 0 to 800 μM over 72 h. All experiments were performed with at least three independent biological replicates unless otherwise specified. Data are represented as mean ± SD. Statistical differences were determined with Student's t-tests (A, B) and one-way ANOVA followed by Tukey’s post hoc test (D, F). Scale bar, 200 μm.

**
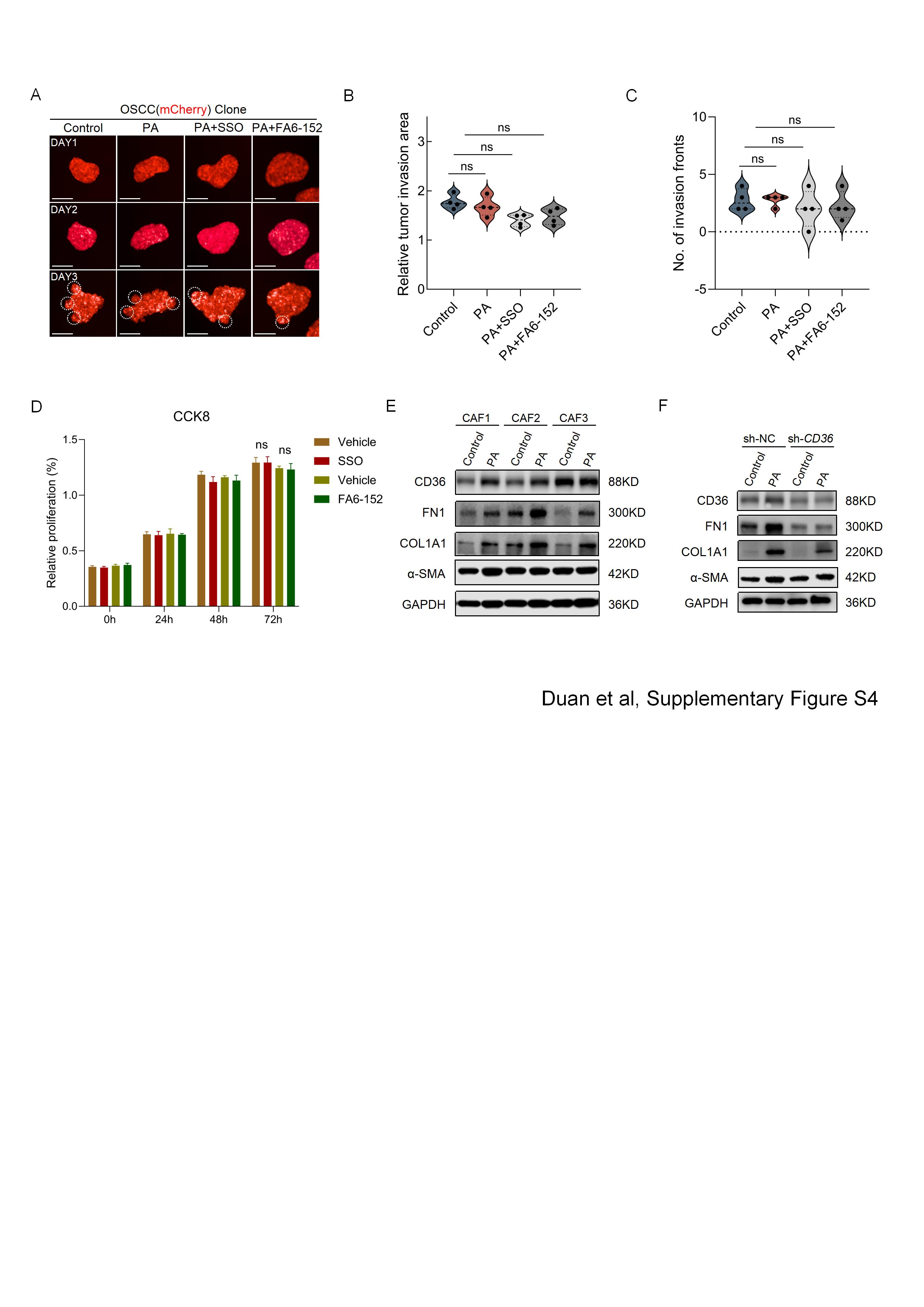
Supplementary Figure S4. PA and CD36 inhibition do not directly affect cancer cell invasion (related to Figure 2).**

(A-C) Control invasion assay using tumor organoids (mCherry-transfected tumor cells cultured alone) to demonstrate that CAF co-culture is required for significant invasion. (A) Representative images and quantification of (B) relative invasion area and (C) number of invasive fronts (n = 4). (D) CCK-8 assay of OSCC cells treated with the respective vehicle controls, SSO, or FA6-152 over 72 h. (E) Western blot analysis demonstrating the expression of α-SMA, FN1, CD36 and COL1A1 in CAFs following PA treatment. (F) Western blot analysis of α-SMA, FN1, CD36 and COL1A1 expression in control and *CD36*-knockdown CAFs with or without PA treatment. All experiments were performed with at least three independent biological replicates unless otherwise specified. Data are represented as mean ± SD. Statistical differences were determined with one-way ANOVA followed by Tukey’s post hoc test (B, C) and unpaired Student's t-tests (D). Scale bar, 200 μm.

**
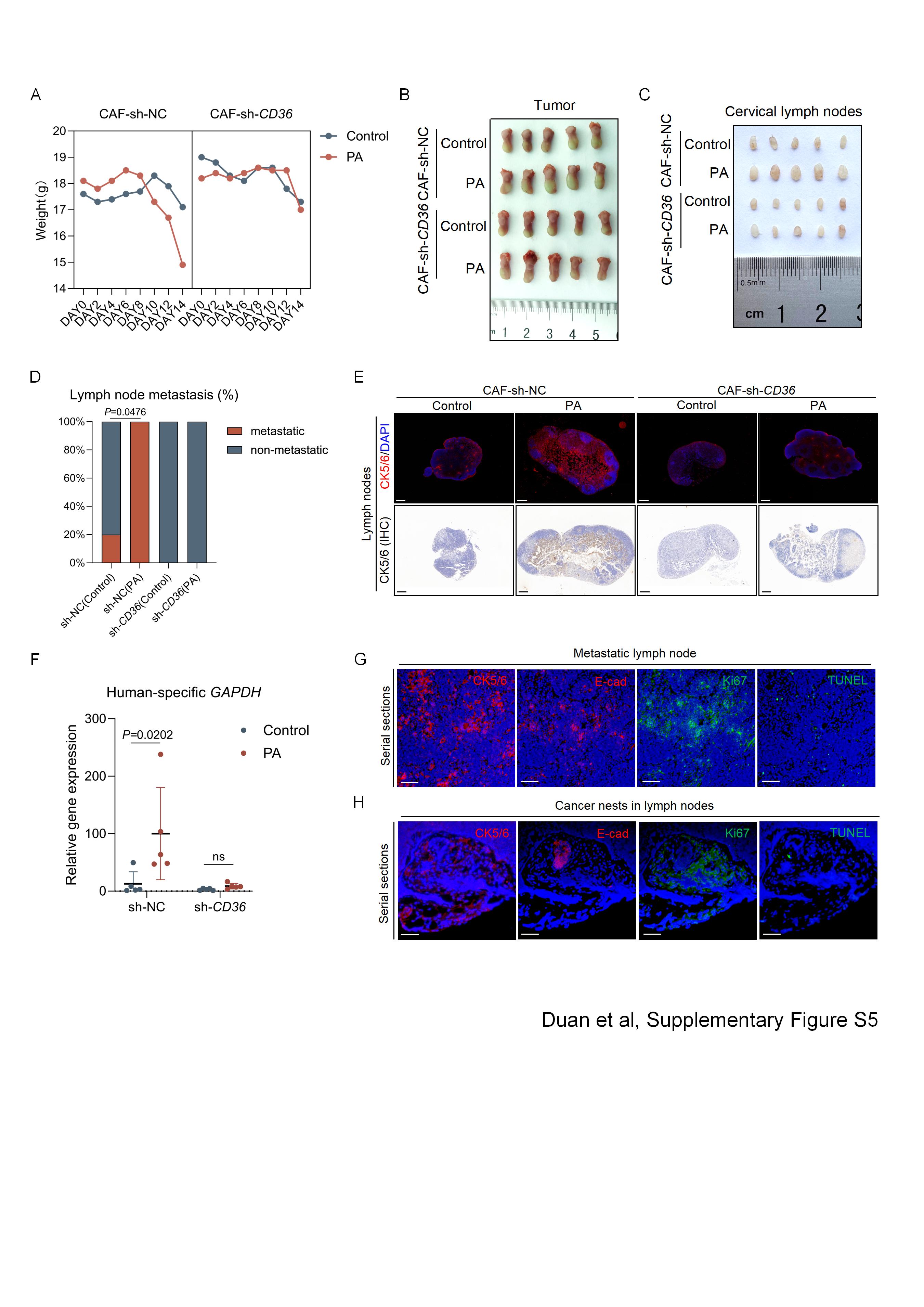
Supplementary Figure S5. CD36 in CAFs contributes to PA-induced metastasis in an orthotopic xenograft model (related to Figure 2).**

(A) Statistical analysis of body weight changes in mice from the experiment described in Figure 2I. (B-C) Photographs of excised primary tongue tumors and cervical lymph nodes from the mice described in Figure 2I (n = 5). (D) Bar graph showing the incidence of lymph node metastasis (percentage of mice with metastatic lymph nodes) in each experimental group. (E) Representative images of CK5/6 and DAPI IF staining in cervical lymph nodes, and CK5/6 immunohistochemistry (IHC) staining of lymph node sections. (F) qPCR analysis of human-specific *GAPDH* expression in cervical lymph nodes from sh-NC and sh-*CD36* groups with or without PA treatment (n = 5). (G) Representative images of CK5/6, E-cadherin, Ki67 and TUNEL staining in serial sections of metastatic lymph nodes. (H) Representative images of CK5/6, E-cadherin, Ki67 and TUNEL staining in serial sections of cancer nests. Data are represented as mean ± SD. Statistical differences were determined with the Chi-square test for (D) and one-way ANOVA followed by Tukey’s post hoc test (F). Scale bar, 200 μm.

**
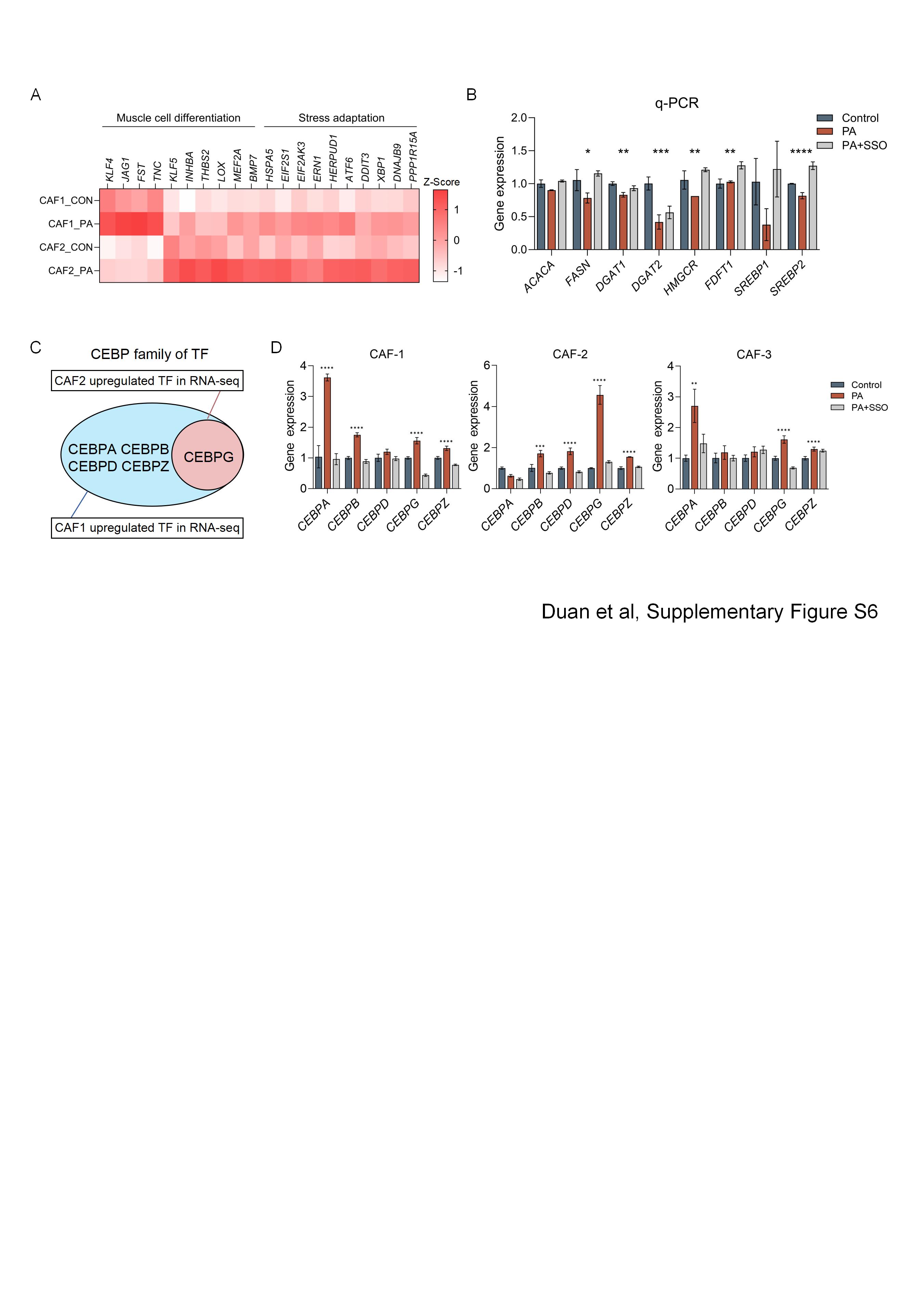
Supplementary Figure S6. Upregulation of *CEBPG* and downregulation of lipid synthesis genes in PA-treated CAFs (related to Figure 3).**

(A) Heatmap showing the expression changes (Z-score) of genes associated with muscle cell differentiation and stress adaptation in two independent CAF lines following PA treatment. (B) qPCR assay of key lipogenic regulators in CAFs (n = 2-3). (C) Venn diagram showing commonly upregulated CEBP-family transcription factors in two independent CAF lines after PA treatment, based on RNA-seq data. (D) qPCR assay of CEBP-family transcription factors in CAFs (n = 3). All experiments were performed with at least three independent biological replicates unless otherwise specified. Data are represented as mean ± SD. Statistical differences were determined with one-way ANOVA followed by Tukey’s post hoc test (B, D). Asterisks denote statistical significance: **P* < 0.05, ***P* < 0.01, ****P* < 0.001, *****P* < 0.0001.

**
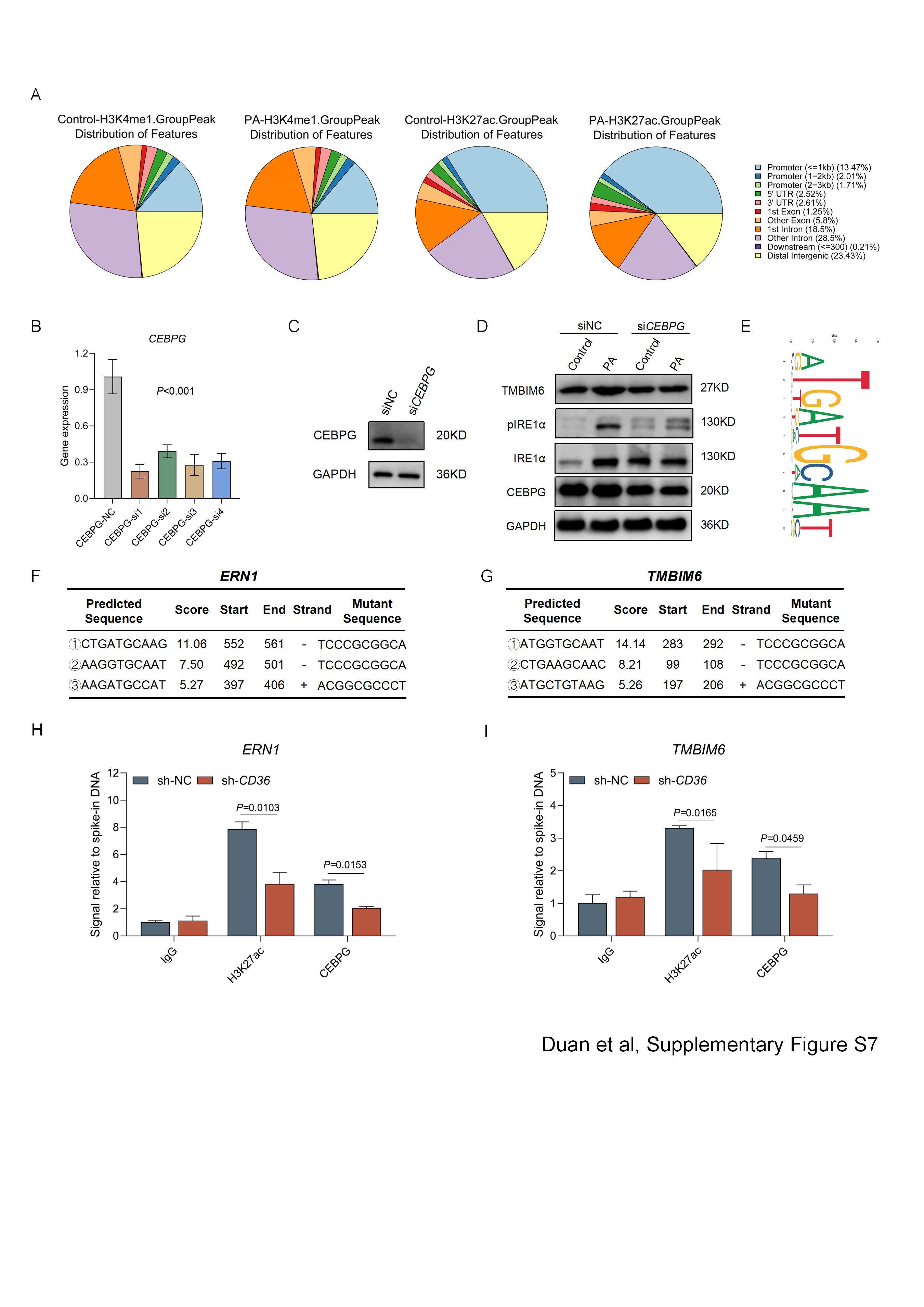
Supplementary Figure S7. Validation of the CEBPG-IRE1α/TMBIM6 axis (related to Figures 4, 5).**

(A) Genome-wide distribution of H3K4me1 and H3K27ac binding peaks in CAFs. (B-C) qPCR and Western blot validation of *CEBPG* knockdown in CAFs. (D) Western blot analysis of CEBPG, TMBIM6, IRE1α, and pIRE1α expression in control and *CEBPG*-knockdown CAFs with or without PA treatment. (E) Sequence logo for the CEBPG binding motif from the JASPAR database. (F-G) The top three sites predicted by the JASPAR database for transcription factor CEBPG binding to enhancer fragments of the *ERN1* and *TMBIM6* genes. (H-I) CUT&RUN-qPCR analysis of H3K27ac enrichment and CEBPG binding at the *ERN1* and *TMBIM6* enhancers in sh-NC and sh-*CD36* CAFs. All experiments were performed with at least three independent biological replicates unless otherwise specified. Data are represented as mean ± SD. Statistical differences were determined with unpaired Student's t-tests (H, I) and one-way ANOVA followed by Tukey’s post hoc test (B).

**
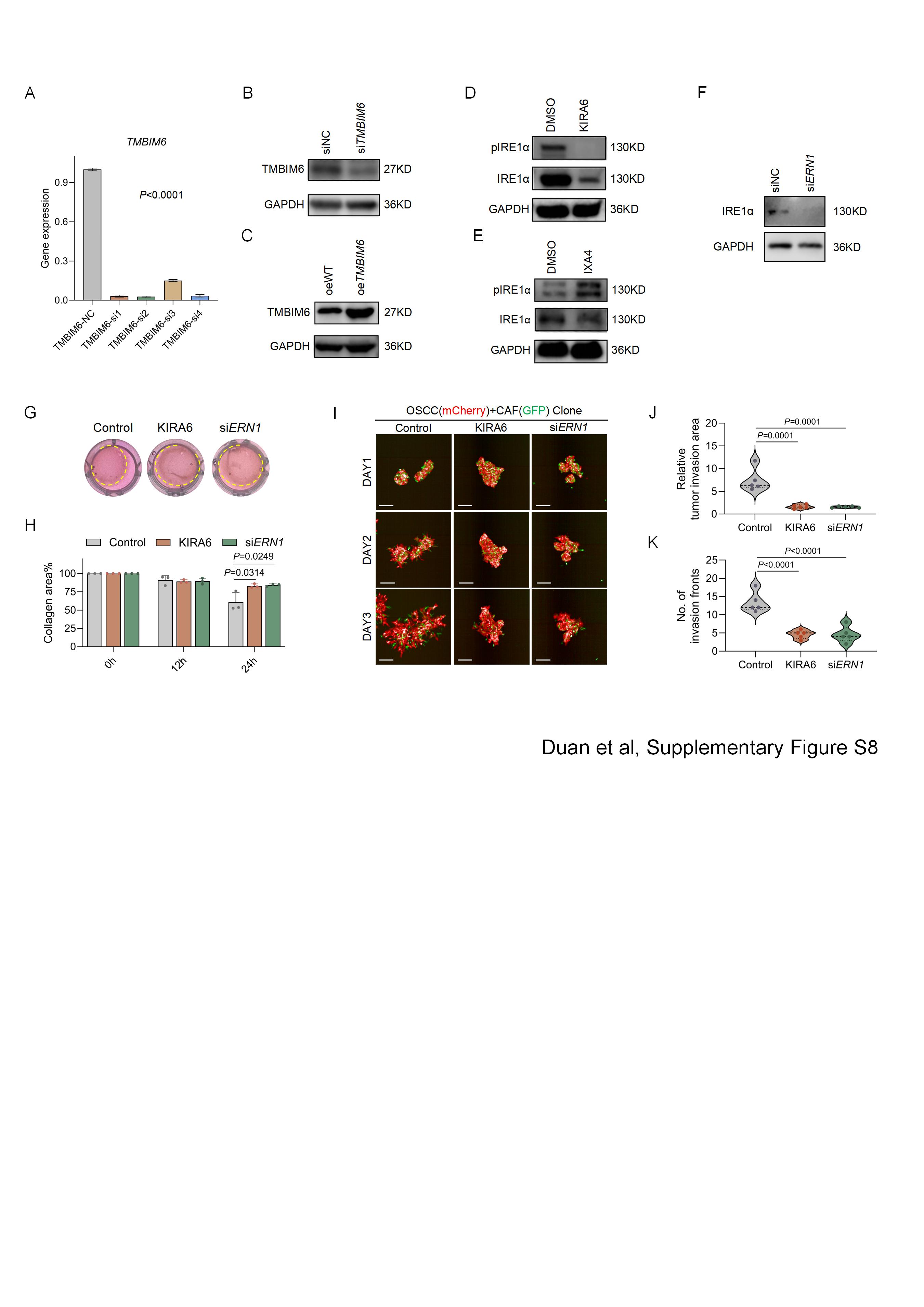
Supplementary Figure S8. Functional validation of TMBIM6 and IRE1α in CAFs (related to Figure 5).**

(A-C) qPCR assay and Western blot validation of *TMBIM6* knockdown and overexpression effects in CAF. (D-E) Western blot validation of IRE1α inhibition by KIRA6 and activation by IXA4 in CAFs. (F) Western blot validation of *ERN1* knockdown (si*ERN1*) efficiency in CAFs. (G) Representative images of collagen contraction assays of CAFs treated with control, KIRA6, or si*ERN1*. (H) Statistical analysis of collagen area percentage at 0, 12, and 24 h (n = 3). (I-K) Representative fluorescence images of FAOs composed of GFP-labeled CAFs (control, KIRA6-treated, or si*ERN1*-treated) and mCherry-labeled tumor cells in a PA environment on day 1 and day 3. Statistical analysis of tumor invasion areas (day 3 vs. day 1) and number of invasion fronts (day 3) in FAOs (n = 5). All experiments were performed with at least three independent biological replicates unless otherwise specified. Data are represented as mean ± SD. Statistical differences were determined with one-way ANOVA followed by Tukey’s post hoc test (A, H, J, K). Scale bar, 200 μm (I).

**
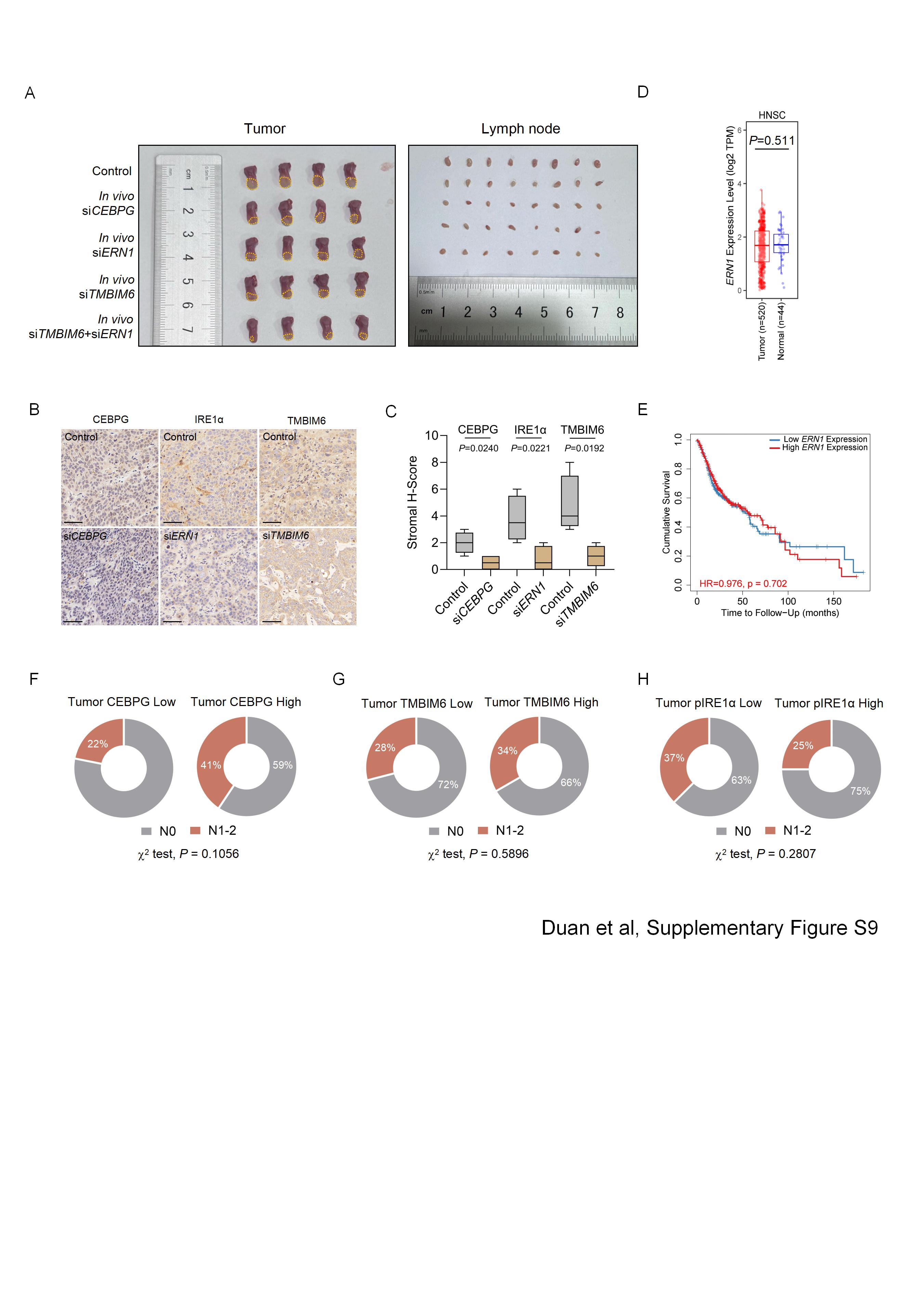
Supplementary Figure S9. *In vivo* validation and clinical relevance of the CEBPG-IRE1α/TMBIM6 axis (related to Figures 5 and 6).**

(A) Photographs of excised primary tongue tumors and cervical lymph nodes from *in vivo* experiment described in Figure 5K-L (n = 4 per group). (B-C) Representative IHC images and statistical analysis of stromal H-scores for CEBPG, IRE1α, and TMBIM6 in orthotopic tongue tumor sections. (D) Gene expression of *ERN1* in HNSC and normal tissues within the TIMER2 database. (E) Kaplan-Meier survival curve for *ERN1* expression in HNSC from the TIMER2 database. (F-H) Statistical analysis and comparison of N stage between groups with high (n = 32) and low expression (n = 32) of tumor CEBPG (F), TMBIM6 (G), and pIRE1α (H). Data are represented as mean ± SD. Statistical significance was determined using Student's t-test for (C, D), the log-rank test for (E), and the Chi-square test for (F-H). Scale bar, 200 μm.

**
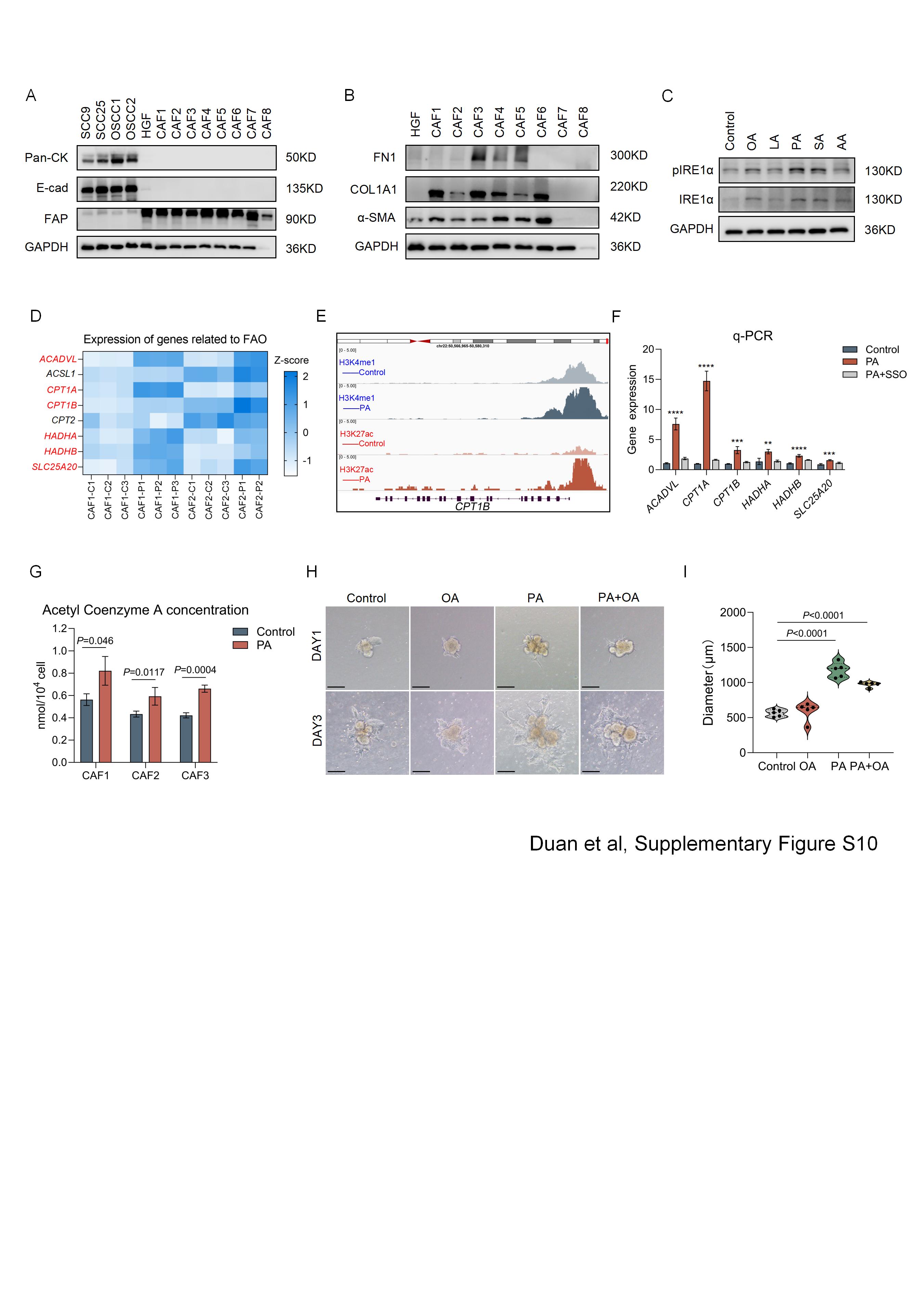
****Supplementary Figure S10. Characterization of primary CAFs and combinatorial fatty acid effects (related to Discussion).**

(A) Western blot analysis of Pan-CK, E-cadherin, FAP, and GAPDH expression in OSCC cell lines (SCC9, SCC25), two independent primary OSCC lines (OSCC1, OSCC2), human gingival fibroblasts (HGF), and eight independent primary CAF lines (CAF1-CAF8). (B) Western blot analysis of FN1, COL1A1, α-SMA, and GAPDH expression in HGF and eight independent primary CAF lines (CAF1-CAF8). (C) Western blot analysis of pIRE1α and IRE1α expression in CAFs treated with control, OA, LA, PA, SA, or AA. (D) Heatmap showing the expression changes of key fatty acid β-oxidation (FAO)-related genes in two independent CAF lines (CAF1 and CAF2) after PA treatment, based on RNA-seq data. (E) IGV tracks showing increased H3K4me1 and H3K27ac occupancy at the CPT1B locus following PA treatment. (F) qPCR assay of FAO-related genes in CAFs (n = 3). (G) Acetyl-CoA concentration in three independent CAF lines (CAF1, CAF2, and CAF3) treated with control or PA. (H-I) Representative bright-field images of FAOs treated with control, OA, PA, or OA+PA on days 1 and 3. Statistical analysis of FAO diameter on day 5 (n = 5). All experiments were performed with at least three independent biological replicates unless otherwise specified. Data are represented as mean ± SD. Statistical differences were determined with unpaired Student's t-tests (G) and one-way ANOVA followed by Tukey’s post hoc test (F, I). Scale bar, 200 μm.

**Supplementary Tables**

**Supplementary Table S1 Clinicopathological information of 90 OSCC samples.**

| No. | Age | Sex | Site | Pathology | TNM Stage^a^ |
| --- | --- | --- | --- | --- | --- |
| 1 | 65 | M | Gingiva | OSCC | T3N0M0 |
| 2 | 33 | M | Palate | OSCC | T4N0M0 |
| 3 | 59 | M | Tongue | OSCC | T2N0M0 |
| 4 | 48 | M | Mouth floor | OSCC | T2N0M0 |
| 5 | 62 | M | Tongue | OSCC | T1N2M0 |
| 6 | 55 | M | Tongue | OSCC | T4N0M0 |
| 7 | 62 | M | Tongue | OSCC | T3N3M0 |
| 8 | 64 | M | Gingiva | OSCC | T1N0M0 |
| 9 | 67 | M | Mouth floor | OSCC | T2N2M0 |
| 10 | 50 | M | Tongue | OSCC | T2N2M0 |
| 11 | 29 | M | Palate | OSCC | T2N2M0 |
| 12 | 38 | M | Tongue | OSCC | T3N0M0 |
| 13 | 37 | M | Tongue | OSCC | T2N0M0 |
| 14 | 60 | M | Mandible | OSCC | T3N2M0 |
| 15 | 37 | M | Tongue | OSCC | T2N2M0 |
| 16 | 41 | M | Tongue | OSCC | T2N0M0 |
| 17 | 26 | M | Cheek | OSCC | T2N0M0 |
| 18 | 55 | M | Cheek | OSCC | T2N2M0 |
| 19 | 36 | M | Tongue | OSCC | T2N0M0 |
| 20 | 59 | M | Cheek | OSCC | T3N1M0 |
| 21 | 61 | M | Tongue | OSCC | T2N0M0 |
| 22 | 69 | F | Gingiva | OSCC | T1N1M0 |
| 23 | 56 | F | Cheek | OSCC | T1N0M0 |
| 24 | 54 | M | Tongue | OSCC | T3N0M0 |
| 25 | 68 | M | Cheek | OSCC | T3N0M0 |
| 26 | 78 | F | Cheek | OSCC | T2N1M0 |
| 27 | 54 | M | Tongue | OSCC | T2N2M0 |
| 28 | 67 | F | Cheek | OSCC | T1N0M0 |
| 29 | 55 | F | Tongue | OSCC | T1N0M0 |
| 30 | 38 | M | Tongue | OSCC | T4N0M0 |
| 31 | 55 | M | Gingiva | OSCC | T2N0M0 |
| 32 | 56 | M | Tongue | OSCC | T2N0M0 |
| 33 | 50 | M | Mouth floor | OSCC | T1N0M0 |
| 34 | 56 | M | Tongue | OSCC | T4N0M0 |
| 35 | 38 | M | Tongue | OSCC | T3N0M0 |
| 36 | 60 | M | Tongue | OSCC | T1N0M0 |
| 37 | 59 | M | Cheek | OSCC | T2N0M0 |
| 38 | 41 | M | Tongue | OSCC | T1N0M0 |
| 39 | 38 | M | Tongue | OSCC | T2N0M0 |
| 40 | 40 | M | Tongue | OSCC | T2N0M0 |
| 41 | 59 | M | Cheek | OSCC | T1N0M0 |
| 42 | 67 | M | Tongue | OSCC | T2N0M0 |
| 43 | 33 | M | Tongue | OSCC | T2N0M0 |
| 44 | 53 | M | Tongue | OSCC | T2N2M0 |
| 45 | 66 | F | Cheek | OSCC | T1N0M0 |
| 46 | 55 | M | Cheek | OSCC | T4N0M0 |
| 47 | 58 | M | Gingiva | OSCC | T1N0M0 |
| 48 | 65 | M | Tongue | OSCC | T3N1M0 |
| 49 | 62 | M | Tongue | OSCC | T2N0M0 |
| 50 | 60 | M | Tongue | OSCC | T2N0M0 |
| 51 | 56 | M | Tongue | OSCC | T2N2M0 |
| 52 | 72 | F | Tongue | OSCC | T2N1M0 |
| 53 | 76 | M | Cheek | OSCC | T2N1M0 |
| 54 | 34 | M | Tongue | OSCC | T2N0M0 |
| 55 | 65 | M | Cheek | OSCC | T4N0M0 |
| 56 | 55 | M | Tongue | OSCC | T2N1M0 |
| 57 | 58 | M | Gingiva | OSCC | T4N0M0 |
| 58 | 60 | M | Tongue | OSCC | T2N1M0 |
| 59 | 39 | M | Tongue | OSCC | T1N0M0 |
| 60 | 63 | M | Cheek | OSCC | T2N0M0 |
| 61 | 50 | F | Tongue | OSCC | T1N0M0 |
| 62 | 62 | M | Mouth floor | OSCC | T4N0M0 |
| 63 | 58 | M | Palate | OSCC | T4N0M0 |
| 64 | 56 | M | Cheek | OSCC | T2N2M0 |
| 65 | 48 | M | Tongue | OSCC | T1N1M0 |
| 66 | 48 | M | Tongue | OSCC | T1N1M0 |
| 67 | 37 | M | Tongue | OSCC | T2N1M0 |
| 68 | 51 | M | Cheek | OSCC | T3N1M0 |
| 69 | 54 | F | Palate | OSCC | T4N0M0 |
| 70 | 37 | M | Mouth floor | OSCC | T4N1M0 |
| 71 | 72 | M | Cheek | OSCC | T4N0M0 |
| 72 | 34 | M | Tongue | OSCC | T1N0M0 |
| 73 | 31 | M | Tongue | OSCC | T1N0M0 |
| 74 | 62 | M | Cheek | OSCC | T2N0M0 |
| 75 | 60 | F | Tongue | OSCC | T2N0M0 |
| 76 | 36 | M | Tongue | OSCC | T2N0M0 |
| 77 | 46 | M | Cheek | OSCC | T2N0M0 |
| 78 | 29 | M | Tongue | OSCC | T2N0M0 |
| 79 | 62 | M | Cheek | OSCC | T2N0M0 |
| 80 | 59 | M | Gingiva | OSCC | T2N0M0 |
| 81 | 32 | M | Cheek | OSCC | T4N1M0 |
| 82 | 54 | M | Cheek | OSCC | T1N0M0 |
| 83 | 29 | M | Cheek | OSCC | T1N0M0 |
| 84 | 43 | M | Tongue | OSCC | T1N0M0 |
| 85 | 65 | M | Pharynx | OSCC | T2N1M0 |
| 86 | 55 | M | Tongue | OSCC | T2N1M0 |
| 87 | 60 | M | Tongue | OSCC | T1N0M0 |
| 88 | 77 | M | Mouth floor | OSCC | T1N0M0 |
| 89 | 48 | F | Tongue | OSCC | T2N1M0 |
| 90 | 55 | M | Cheek | OSCC | T2N2M0 |

^a^TNM staging was defined according to the American Joint Committee on Cancer (AJCC) 7th edition.

OSCC, oral squamous cell carcinoma; M, male; F, female.

**Supplementary Table S2 Sequences of siRNA.**

| siRNA | Sense (5' to 3') | Antisense (5' to 3') |
| --- | --- | --- |
| siCEBPG | GAUCGAAACAGUGACGAGUTT | ACUCGUCACUGUUUCGAUCTT |
| siTMBIM6 | GCCUAUGUCCAUAUGGUCATT | UGACCAUAUGGACAUAGGCTT |
| siERN1 | CUCCGAGCCAUGAGAAAUATT | UAUUUCUCAUGGCUCGGAGTT |

**Supplementary Table S3** **Sequences of qPCR primers.**

| Gene | Forward Primer (5' to 3') | Reverse Primer (5' to 3') |
| --- | --- | --- |
| *HSPA5* | GAAAGAAGGTTACCCATGCAGT | CAGGCCATAAGCAATAGCAGC |
| *EIF2S1* | TGGTGAATGTCAGATCCATTGC | TAGAACGGATACGCCTTCTGG |
| *EIF2AK3* | GGAAACGAGAGCCGGATTTATT | ACTATGTCCATTATGGCAGCTTC |
| *ERN1* | CACAGTGACGCTTCCTGAAAC | GCCATCATTAGGATCTGGGAGA |
| *ATF4* | ATGACCGAAATGAGCTTCCTG | GCTGGAGAACCCATGAGGT |
| *ATF6* | AGCAGCACCCAAGACTCAAAC | GCATAAGCGTTGGTACTGTCTGA |
| *DDIT3* | GGAAACAGAGTGGTCATTCCC | CTGCTTGAGCCGTTCATTCTC |
| *ACADVL* | GGAAGCTCGCGGCTCA | TTTTCCTGGTCAGAGCGTCA |
| *CPT1A* | CTCAGTGGGAGCGGATGTTT | TGCTGTCTCTCATGTGCTGG |
| *CPT1B* | GGGGTAAGTTCTGCCTGACC | ACAGGAACGCACAGTCTCAG |
| *HADHA* | AGACCGAGGACAGCAACAAG | GCTTCAATCACCATGTCGGC |
| *HADHB* | CCCTGCGGTTTCTGAGTTCT | TCCTGTTCCAGCCGAGAAAC |
| *SLC25A20* | AATGGCTGCCCCTATCATCG | GCTGAGCACATCTTCTGGGT |
| *CEBPA* | TAACTCCCCCATGGAGTCGG | TGTCGATGGACGTCTCGTG |
| *CEBPB* | CTTCAGCCCGTACCTGGAG | GGAGAGGAAGTCGTGGTGC |
| *CEBPD* | CCATGTACGACGACGAGAGC | GTTGAAGAGGTCGGCGAAGA |
| *CEBPG* | ACTCCAGGGGTGAACGGAAT | CATGGGCGAACTCTTTTTGCT |
| *CEBPZ* | TGATGCCGTTCACACACTTCA | CAAGGCCATAAGGCACTGC |
| *TMBIM6* | CATATAACCCCGTCAACGCAG | GCAGCCGCCACAAACATAC |
| *ACACA* | ATGTCTGGCTTGCACCTAGTA | CCCCAAAGCGAGTAACAAATTCT |
| *FASN* | AAGGACCTGTCTAGGTTTGATGC | TGGCTTCATAGGTGACTTCCA |
| *SCD1* | TCTAGCTCCTATACCACCACCA | TCGTCTCCAACTTATCTCCTCC |
| *DGAT1* | TATTGCGGCCAATGTCTTTGC | CACTGGAGTGATAGACTCAACCA |
| *DGAT2* | ATTGCTGGCTCATCGCTGT | GGGAAAGTAGTCTCGAAAGTAGC |
| *HMGCR* | TGATTGACCTTTCCAGAGCAAG | CTAAAATTGCCATTCCACGAGC |
| *FDFT1* | CCACCCCGAAGAGTTCTACAA | TGCGACTGGTCTGATTGAGATA |
| *SREBF1* | ACAGTGACTTCCCTGGCCTAT | GCATGGACGGGTACATCTTCAA |
| *SREBF2* | CCTGGGAGACATCGACGAGAT | TGAATGACCGTTGCACTGAAG |
| *GAPDH (human-specific* | TCAAGGCTGAGAACGGGAAG | CGCCCCACTTGATTTTGGAG |
| *B2m* | TTCTGGTGCTTGTCTCACTGA | CAGTATGTTCGGCTTCCCATTC |

**Supplementary Table S4 Antibodies utilized in this study.**

| Antibody | Resource | Identifier | Application |
| --- | --- | --- | --- |
| α-SMA | Cell Signaling Technology | Cat# 19245, RRID: AB_2734735 | WB, IF |
| FN1 | Cell Signaling Technology | Cat# 26836, RRID: AB_2924220 | WB, IF |
| COL1A1 | Cell Signaling Technology | Cat# 72026, RRID: AB_2904565 | WB, IF |
| IRE1α | Proteintech | Cat# 27528-1-AP, RRID: AB_2880899 | WB |
| TMBIM6 | Proteintech | Cat# 26782-1-AP, RRID: AB_2880633 | WB, IF |
| CEBPG | Proteintech | Cat# 12997-1-AP, RRID: AB_2877902 | WB, IF |
| CD36 | Abcam | Cat# ab252922, RRID: AB_2922821 | WB, IF, IHC |
| Phospho-IRE1α(S724) | HUABIO | Cat# HA721980, RRID: AB_3096844 | WB, IF |
| F-actin | Abcam | Cat# ab205, RRID: AB_302794 | IF |
| PDGFRA | Cell Signaling Technology | Cat# 5241, RRID: AB_10692773 | IF |
| PDGFRB | Cell Signaling Technology | Cat# 3169, RRID: AB_2162497 | IF |
| CK5/6 | Proteintech | Cat# 68295-1-Ig, RRID: AB_2935375 | IF |
| Pan-Keratin | Cell Signaling Technology | Cat# 4545, RRID: AB_2162497 | WB |
| FAP | Cell Signaling Technology | Cat# 66562, RRID: AB_2904193 | WB |
| E-Cadherin | Cell Signaling Technology | Cat# 3195, RRID: AB_2291471 | WB, IF |
| Ki67 | Cell Signaling Technology | Cat# 9129, RRID: AB_2687446 | IF |
| H3K4me1 | Cell Signaling Technology | Cat# 5326, RRID: AB_10695148 | CUT&Tag, CUT&RUN |
| H3K27ac | Cell Signaling Technology | Cat# 8173, RRID: AB_10949503 | CUT&Tag, CUT&RUN |
| IgG | Cell Signaling Technology | Cat# 2729, RRID: AB_1031062 | CUT&Tag |
| Goat Anti-Rabbit IgG (H+L) | Cell Signaling Technology | Cat# 35401, RRID: AB_3683670 | CUT&Tag |
| CEBPG | Thermo Fisher Scientific | Cat# PA5-80468, RRID: AB_2787782 | CUT&RUN |
| mAb IgG | Cell Signaling Technology | Cat# 66362, RRID: AB_2924329 | CUT&RUN |
| CD36 | Abcam | Cat# ab17044, RRID: AB_443600 | Neutralization |

WB: Western blot; IF: immunofluorescence; IHC: immunohistochemistry; CUT&Tag: Cleavage Under Targets and Tagmentation; CUT&RUN: Cleavage Under Targets and Release Using Nuclease.

**Supplementary Table S5 Sequences of primers for CUT&RUN-qPCR.**

| Target Locus | Forward Primer (5' to 3') | Reverse Primer (5' to 3') |
| --- | --- | --- |
| *ERN1* | CATGCAAGCCAAGAAGAGCA | TGGCCATGTCCTCTTAATCTGT |
| *TMBIM6* | TATGGAAGGCTGTGGTTGGC | CTCTGAGTCACACGGATCTGG |
